# Global priorities for conservation of reptilian phylogenetic diversity in the face of human impacts

**DOI:** 10.1038/s41467-020-16410-6

**Published:** 2020-05-26

**Authors:** Rikki Gumbs, Claudia L. Gray, Monika Böhm, Michael Hoffmann, Richard Grenyer, Walter Jetz, Shai Meiri, Uri Roll, Nisha R. Owen, James Rosindell

**Affiliations:** 1 Department of Life Sciences, Imperial College London, Silwood Park Campus, Ascot, Berkshire, SL5 7PY UK; 2 Science and Solutions for a Changing Planet DTP, Grantham Institute, Imperial College London, Exhibition Road, South Kensington, London, SW7 2AZ UK; 3 EDGE of Existence Programme, Zoological Society of London, Regent’s Park, London, NW1 4RY UK; 4 Institute of Zoology, Zoological Society of London, Regent’s Park, London, NW1 4RY UK; 5 Conservation and Policy, Zoological Society of London, Regent’s Park, London, NW1 4RY UK; 6 School of Geography and the Environment, University of Oxford, Oxford, OX1 3QY UK; 7 Ecology and Evolutionary Biology Department, Yale University, 165 Prospect Street, New Haven, CT 06511 USA; 8 Center for Biodiversity and Global Change, Yale University, 165 Prospect Street, New Haven, CT 06511 USA; 9 School of Zoology, Tel Aviv University, 6997801 Tel Aviv, Israel; 10 Steinhardt Museum of Natural History, Tel Aviv University, 6997801 Tel Aviv, Israel; 11 Mitrani Department of Desert Ecology, Ben-Gurion University of the Negev, Midreshet Ben-Gurion, 8499000 Israel; 12 On The EDGE Conservation, 152a Walton St, Chelsea, London, SW3 2JJ UK

**Keywords:** Biodiversity, Conservation biology, Phylogenetics, Herpetology

## Abstract

Phylogenetic diversity measures are increasingly used in conservation planning to represent aspects of biodiversity beyond that captured by species richness. Here we develop two new metrics that combine phylogenetic diversity and the extent of human pressure across the spatial distribution of species — one metric valuing regions and another prioritising species. We evaluate these metrics for reptiles, which have been largely neglected in previous studies, and contrast these results with equivalent calculations for all terrestrial vertebrate groups. We find that regions under high human pressure coincide with the most irreplaceable areas of reptilian diversity, and more than expected by chance. The highest priority reptile species score far above the top mammal and bird species, and reptiles include a disproportionate number of species with insufficient extinction risk data. Data Deficient species are, in terms of our species-level metric, comparable to Critically Endangered species and therefore may require urgent conservation attention.

## Abstract (web summary)

In addition to species richness, evolutionary measures of biodiversity are important considerations for conservation. Here, Gumbs et al. develop new biodiversity metrics incorporating phylogenetic diversity and human pressure and highlight conservation priorities in a global analysis of reptiles.

## Introduction

We are in the midst of a global biodiversity crisis^1,2^ with severely limited resources for conservation action^3^. At current extinction rates, we are set to experience unprecedented losses of species and their phylogenetic diversity (PD). PD is the sum of the phylogenetic branch lengths connecting a set of species to each other across their phylogenetic tree, and measures their collective contribution to the tree of life^4,5^. PD quantifies the amount of evolutionary variation across a set of species^4^, and is thus a valuable tool for prioritising species and regions for conservation^5–8^.

PD is increasingly recognised as an important component of global biodiversity^9,10^ linked to increased ecosystem productivity^11,12^ and human well-being^4,13,14^. The International Union for the Conservation of Nature (IUCN) recognises the importance of conserving PD (in the forms of ‘taxonomic hierarchy’^15^ and ‘evolutionarily distinct lineages’^16^) and has established a Task Force of the Species Survival Commission dedicated to PD conservation^17^. Similarly, the Intergovernmental Science-Policy Platform on Biodiversity and Ecosystem Services (IPBES) recognises PD as a key indicator of global trends in nature’s contribution to people^18^.

Prioritising species which represent large amounts of unique PD can potentially conserve more phylogenetic and trait diversity than phylogenetically uninformed prioritisations^19,20^, though the relationship between PD and trait diversity is complex and variable^20^. Phylogenetically informed prioritisations are used to direct conservation efforts on the ground, the most notable example of which is the Zoological Society of London’s EDGE of Existence programme. The programme has implemented more than 110 conservation projects on threatened species comprising large amounts of unique PD^6,21^.

Many studies have explored how PD can be conserved across mammals and birds^5,6,10,22–25^. Reptiles (crocodilians, turtles, squamates (i.e. lizards and snakes), and the tuatara, but excluding birds) remain poorly studied in global conservation schemes^26^ despite comprising ~30% of terrestrial vertebrate species^27^. Around 60% of the world’s turtles^28^, almost 50% of all crocodilians^29^ and nearly 20% of assessed lizards and snakes are threatened with extinction^30^. Reptile populations have suffered average global declines of around 55% between 1970 and 2012^31^. Existing protected areas and global conservation schemes represent reptiles poorly compared with birds and mammals^32^. Consequently, there is a pressing need to assess all reptiles to enable targeted conservation and allow their incorporation into global conservation prioritisations.

There are several methods available for mapping imperiled PD^8,10,22,33,34^ and, in lieu of explicit extinction risk data, range-restricted species have often been used to identify regions of high conservation value^8,22^. Phylogenetic endemism (PE)^8^ and evolutionary distinctiveness rarity (EDR)^22^ weight branches of the phylogeny by the range sizes of the descendant species to identify regions containing large amounts of PD restricted to small areas. These methods prioritise highly irreplaceable regions but do not incorporate spatial measures of vulnerability, such as human impact, limiting their practical application in conservation planning^35,36^. Unfortunately, while range data are now available for 99% of reptiles^32^, up-to-date extinction risk data (i.e. published in the past 10 years^29,37^) are not yet available for all reptile species^29^. Without comprehensive extinction risk assessments for all reptiles, range data can be combined with environmental data to determine spatial vulnerability^38–40^.

The Human Footprint index (HF)^41,42^ is the most comprehensive and high-resolution dataset of human pressures on global environments. It combines eight variables which measure direct human impacts on the environment, such as agricultural land, built environments, and human population density^42^. Maps of cumulative human pressures have been shown to predict species distributions better than biological traits^43^ and are a strong predictor of species extinction risk^44^. However, the Human Footprint index has not been used to value and prioritise the conservation of terrestrial vertebrate PD globally.

Here, we present three new metrics, two of which combine human pressure (to measure vulnerability), PD and range size (to measure irreplaceability). (1) Our spatial metric, human-impacted phylogenetic endemism (HIPE), is an extension of standard PE that weighs phylogenetic branches in space in relation to the level of human pressure across the range of each species. We use HIPE to identify high value regions that support irreplaceable reptilian PD. We also develop two species-level metrics. (2) Terminal endemism (TE) weights the unique contribution of each species to global PD—the terminal branch length (TBL)—by its range size. (3) Human-impacted terminal endemism (HITE) extends TE by weighting the TBL of each species by the human pressure across its range. We use HITE to identify priority species with small ranges, heavily impacted by humans, whose conservation would safeguard significant amounts of unique PD. We calculated these metrics for all tetrapod clades globally.

Our analyses reveal that concentrations of highly irreplaceable reptilian diversity coincide with elevated human pressure. This diversity is concentrated in highly impacted regions across much of South and East Asia and the Caribbean. Together, reptiles harbour more human-impacted diversity per grid cell globally than amphibians, birds or mammals. At the species level, amphibians have the greatest levels of unique PD under high human pressure. Finally, the lack of extinction risk data for reptiles obscures the true extent of threatened PD, with potentially billions of additional years of PD at risk.

## Results

### Spatial value metric for conserving PD

We find that small ranges—key determinants of extinction risk—are phylogenetically clumped in lepidosaurs (Pagel’s test for phylogenetic signal: λ = 0.373, *p* ≪ 0.0001; Supplementary Fig. 1), and for lizards, amphisbaenians, and the tuatara (hereafter collectively ‘lizards’) and snakes independently (lizards: λ = 0.485, *p* ≪ 0.0001; snakes: λ = 0.345, *p* ≪ 0.0001), but not in turtles (λ = 0.12, *p* = 0.03) or crocodilians (λ = 0.048, *p* = 0.815; following correction for multiple testing, adjusted *p*-value threshold = 0.01).

**Fig. 1:**
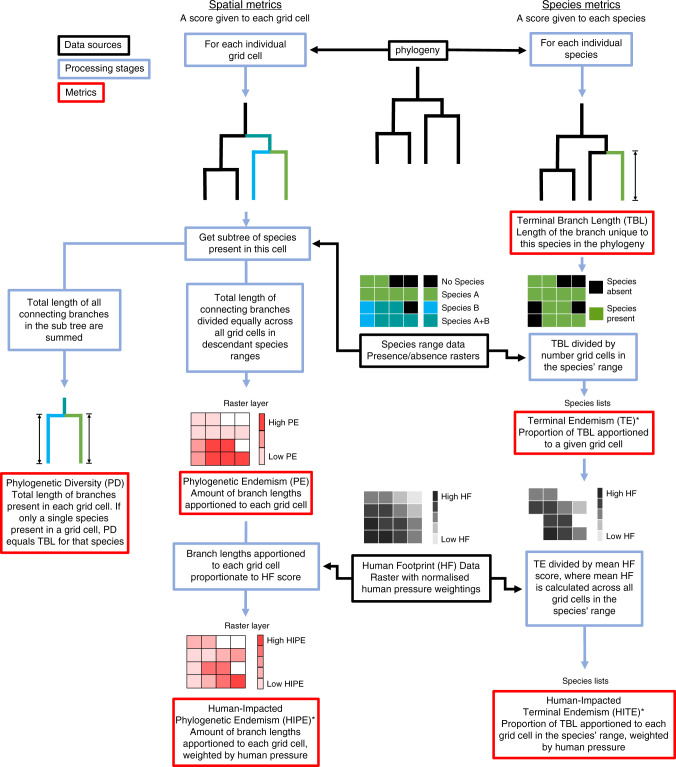
Relationships between spatial and species-level phylogenetic diversity-based metrics. Flowchart illustrating the data sources used, stages of processing, and relationships between the spatial and species-level metrics used here. Metrics are in red boxes, data sources in black, and processing stages in blue. Only those metrics for which results are reported and presented in the main text are included. Our three novel metrics are denoted with asterisks. Mathematical definitions for all spatial and species-level metrics are discussed in Supplementary Table 1.

**Fig. 2:**
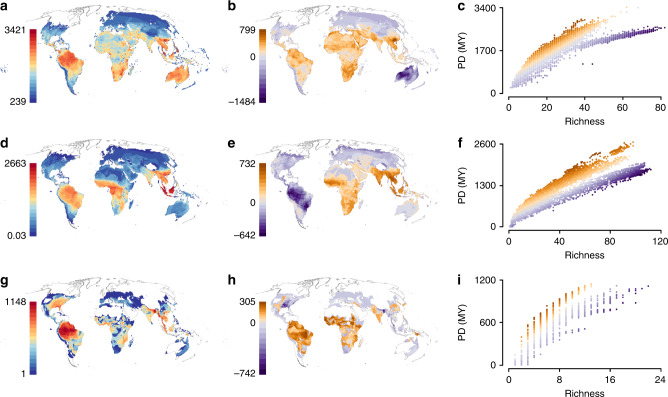
Global patterns of reptilian phylogenetic diversity (PD). Cumulative PD, in millions of years (MY) (left), Middle: residual PD per grid cell, in MY, (warm colours: more than expected given richness, cold colours: less than expected given richness), Right: the relationship between richness and PD across all grid cells from the middle panels for lizards (**a**–**c**), snakes (**d**–**f**), and turtles (**g**–**i**), with colours corresponding to the grid cell values from the middle panels.

We used published and novel PD-based metrics to explore spatial patterns of irreplaceable and vulnerable reptilian PD (Fig. 1, Supplementary Table 1). Global reptilian PD is largely concentrated throughout the tropics (Fig. 2, Supplementary Fig. 2), and is strongly correlated with species richness (Pearson’s correlation: *r* = 0.952, e.d.f. = 23.7, *p* ≪ 0.0001; all correlations spatially corrected), lizards (*r* = 0.920, e.d.f. = 21.1, *p* ≪ 0.0001), snakes (*r* = 0.899, e.d.f. = 24.4, *p* ≪ 0.0001) and turtles (*r* = 0.873, e.d.f. = 28.1, *p* ≪ 0.0001). Lizard PD is highest across Southeast Asia, the Amazon basin and Australia (Fig. 2a). Snake PD is mainly concentrated in Malaysia and Indonesia (Fig. 2d), and turtle PD is mainly concentrated in the Amazon Basin (Fig. 2g), despite turtle richness peaking in the Ganges Delta.

The greatest levels of lizard PD, when corrected for species richness (see Methods), are in mainland Southeast Asia, whereas regions with the lowest levels of residual PD occur across Australia, where richness is highest (Fig. 2b, c). The highest snake PD for a given richness occurs in mainland Southeast Asia, and the lowest coincides with the species-rich Amazon Basin and Atlantic coast of Brazil (Fig. 2e, f). The highest turtle PD for a given richness occurs across subtropical West and Central Africa and the Amazon Basin, and is lowest where species richness peaks: the Ganges Delta and Eastern USA (Fig. 2h, i).

Two existing metrics for mapping irreplaceable PD: PE and EDR (Supplementary Table 1) are highly correlated for reptiles globally (spatially corrected correlation: *r* = 0.975, e.d.f. = 537, *p* ≪ 0.0001) and both are highly correlated with the non-phylogenetic measure of Weighted Endemism (WE; both *r* > 0.93; Supplementary Fig. 3). Because PE takes into account the spatial complementarity of species distributions—whereas EDR does not—we think that PE better reflects the impacts of landscape-level threats than EDR and therefore employ PE in our analyses accounting for human pressure hereafter (see Methods).

To determine the extent of human pressure on regions of irreplaceable reptilian diversity, we explored the relationship between the Human Footprint index^42^ and reptilian PE globally. We find that PE and Human Footprint are positively correlated globally (spatially corrected correlation: *r* = 0.16, e.d.f. = 514.011, *p* < 0.001). Regions containing the two highest pressure categories (‘high’ and ‘very high’, with Human Footprint ≥ 6 and ≥ 12, respectively^42^) harbour significantly greater amounts of reptilian PE than categories of lower human pressure (Tukey HSD < 0.05).

**Fig. 3:**
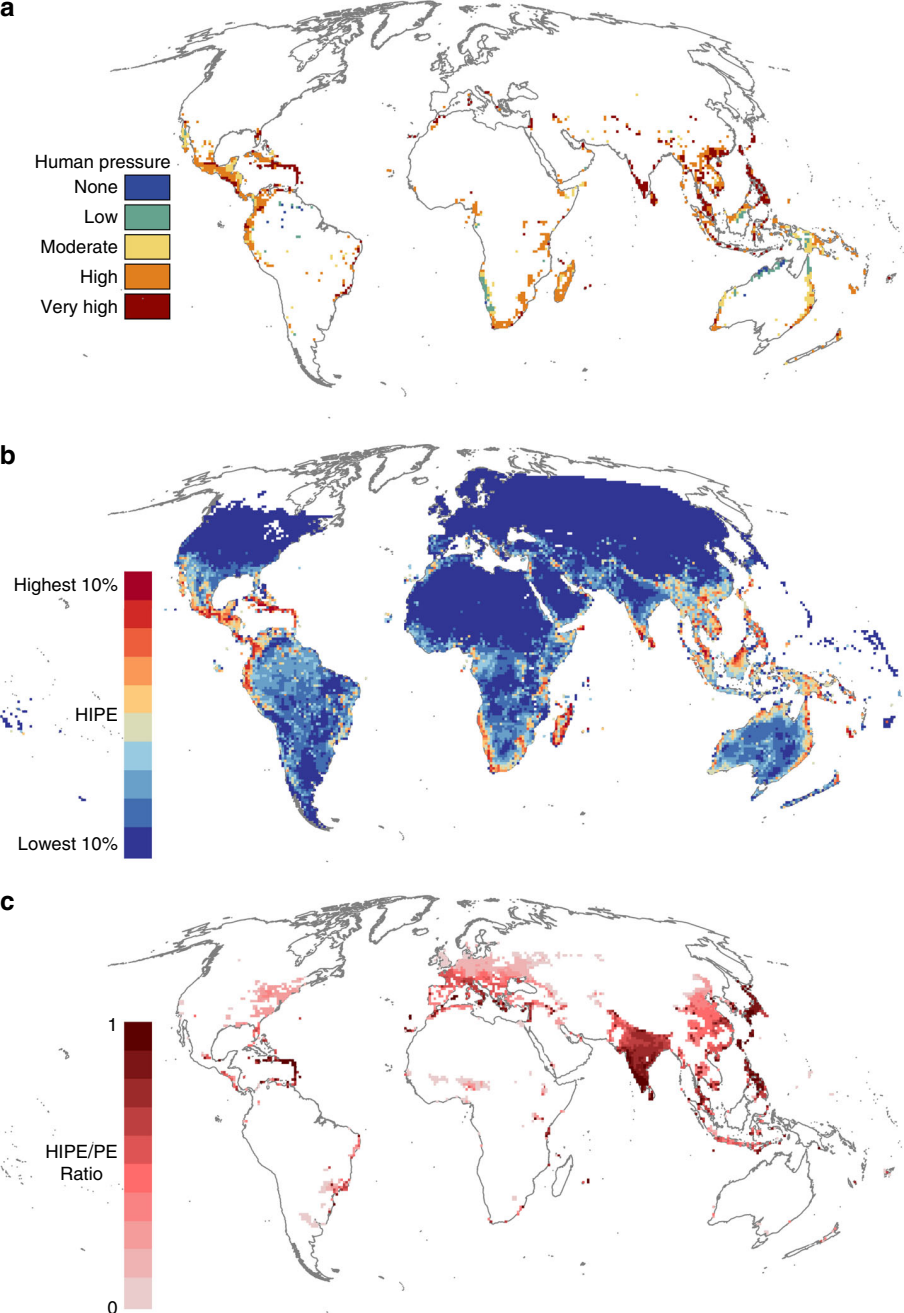
Global patterns of reptilian phylogenetic endemism (PE) and human pressure. **a** regions of high reptilian PE (richest 10% of grid cells) and the level of human pressure in each grid cell. **b** Global patterns of reptilian human-impacted phylogenetic endemism (HIPE), where grid cells are coloured by the cumulative amount of global HIPE captured; darkest red cells comprise the highest-scoring grid cells which together capture 10% of global HIPE, whereas the lowest-scoring grid cells which together capture 10% of global HIPE are coloured dark blue. **c** ratio of HIPE to PE for grid cells under very high human pressure (HF ≥ 12). Given that we only show cells of very high human pressure, a ratio of 1:1 (darkest red) means all PD found in the grid cell is restricted to regions of very high human pressure, and the lower this becomes (increasingly lighter reds), the greater the proportion of phylogenetic diversity also distributed in regions with less human pressure.

Regions harbouring high concentrations of irreplaceable diversity are of great importance for the conservation of biodiversity. We follow Venter et al.^42^ in identifying the richest 10% of global reptilian PE as high value regions. We find that almost three-quarters (74%) of these high PE grid cells are in regions of high or very high human pressure (Human Footprint ≥ 6, Fig. 3a). Conversely, just 5% of these high value grid cells coincide with regions of low or no human pressure (HF < 3, Fig. 3a). This is a greater coincidence of high value regions for biodiversity and high human pressure than we would expect if human pressure was distributed randomly. When we randomise the distribution of human pressure across all grid cells in which reptiles occur, less than half (49%) of high-value grid cells coincide with high or very high human pressure, and 20% coincide with regions of low or no human pressure.

High reptilian PE coincides with very high human pressure (HF ≥ 12) across the tropics—particularly in India, Caribbean islands, the Atlantic Coast of Brazil and Southeast Asia—the Mediterranean coast and areas of the Middle East (Fig. 3a). Regions of high PE and no human pressure (HF < 1) are largely restricted to the Amazon Basin, Namib coast and northern Australia (Fig. 3a).

Our combined measure of human pressure and PE, HIPE, is an extension of standard PE which, rather than distributing the PD of phylogenetic branches evenly across space, distributes PD in relation to the level of human pressure exerted across the distribution (i.e., is weighted against highly impacted areas; Fig. 1, Supplementary Table 1). Consequently, branches distributed across grid cells of both high and low human pressure will have a larger proportion of their PD allocated to grid cells with lower human pressure, under the assumption that grid cells under lower human pressure are more valuable (and favourable) for species persistence.

HIPE is correlated with standard PE for reptiles across all grid cells globally (spatially corrected correlation: *r* = 0.978, e.d.f. = 448, *p* ≪ 0.0001; Supplementary Fig. 3), despite individual grid cell values differing from PE by up to 165% (median = 6%). When we compare the spatial distribution of grid cells comprising the richest 10% of global reptilian PE (Fig. 3a) with those comprising the richest 10% of HIPE (darkest red grid cells, Fig. 3b), there is a 90.4% overlap in grid cell coverage. Of the 10% of richest HIPE grid cells 51% increase in importance relative to PE, including 130 grid cells (9.6%) that are not present in the richest 10% of PE grid cells.

When a branch is distributed entirely across grid cells with the same level of human pressure (e.g. very high pressure; see Methods), its PD is divided equally amongst each grid cell under both HIPE and PE. However, when a branch is distributed across grid cells of differing human pressure, the grid cells with lower human pressure receive a greater proportion of the branch’s PD under HIPE than they do under PE. Conversely, grid cells in the distribution with higher human pressure (e.g. very high) receive a smaller proportion of the branch’s PD under HIPE than they do under PE.

We can therefore use the ratio of HIPE to PE for grid cells experiencing the highest level of human pressure (very high; HF ≥ 12) to identify regions where large proportions of the PD present is under very high human pressure. These are represented by grid cells under very high human pressure and where the ratio of HIPE to PE approaches 1, indicating the PD is largely being divided equally among grid cells with very high human pressure. The Western Ghats, large parts of the Caribbean, the Philippines, Japan, and the Mediterranean harbour reptilian PD that is overwhelmingly restricted to regions of very high human pressure (HIPE/PE ratio > 0.9, Fig. 3c).

The ratio of HIPE to PE for grid cells under the lowest human pressure (HF = 0) can indicate intact regions of the planet that harbour diversity under little or no impact globally when the ratio of HIPE to PE in these grid cells approaches 1. For reptiles these include the Amazon Basin, South Pacific islands, and Central and Northern Australia (Supplementary Fig. 4).

Globally, reptilian HIPE is greatest in Madagascar, Central America and the Caribbean, the Western Ghats, Sri Lanka, Socotra, peninsular Malaysia and northern Borneo (Fig. 3b). Global patterns of lizard HIPE largely reflect those of all reptiles (Supplementary Fig. 5), whereas those for snakes emphasise Central Africa and Southeast Asia (Supplementary Fig. 5). High levels of turtle HIPE are concentrated in Central America, the Atlantic Coast of Brazil, the Western Ghats, Southeast Asia, New Guinea, and northern Australia (Supplementary Fig. 5).

Grid cells have greater median and maximum HIPE scores for lepidosaurs than for other tetrapods (median = 5 × 10^−4^ MY^−1^ km^2^ vs turtles = 1.2 × 10^−4^ MY^−1^ km^2^, amphibians = 4.2 × 10^−4^ MY^−1^ km^2^, birds = 4.3 × 10^−4^ MY^−1^ km^2^, mammals = 3.6 × 10^−4^ MY^−1^ km^2^; maxima: lepidosaurs = 0.37 MY^−1^ km^2^ vs turtles = 0.02 MY^−1^ km^2^, amphibians = 0.30 MY^−1^ km^2^, birds = 0.05 MY^−1^ km^2^, mammals = 0.03 MY^−1^ km^2^). We repeated these analyses with all phylogenetic trees rarefied to 75.5% (lowest percentage of species with phylogenetic range data for a clade, i.e. the Amphibia, Supplementary Table 2) of taxonomic completeness to account for differential sampling across taxonomic groups and our results remained unchanged, with lepidosaurs having the greatest grid cell values of HIPE across all clades (Supplementary Table 3).

**Fig. 4:**
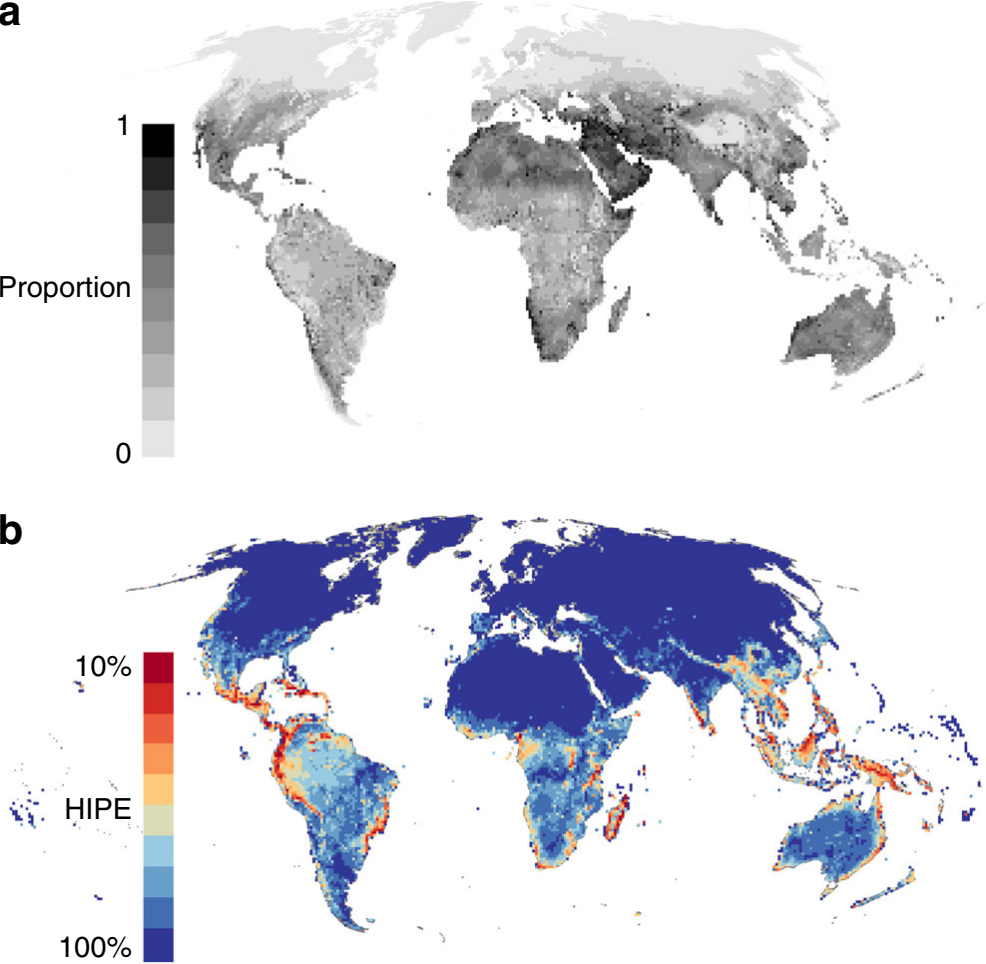
Global patterns of tetrapod human-impacted phylogenetic endemism (HIPE) and reptilian contributions. The global patterns of (**a**) the proportion of tetrapod HIPE contributed by reptiles (from 100% of HIPE contributed by reptiles in black to 0% of HIPE in light grey); and (**b**) tetrapod HIPE, where grid cells are coloured by the cumulative amount of global HIPE captured; darkest red cells comprise the highest-scoring grid cells which together capture 10% of global HIPE, whereas the lowest-scoring grid cells which together capture 10% of global HIPE are coloured dark blue.

When combined, reptiles contribute a median of 31.1% to tetrapod HIPE scores across all grid cells in which they are present, more than any other tetrapod group (lepidosaurs = 27%, turtles = 3%, amphibians = 16.6%, birds = 29.7%, mammals = 18%; Supplementary Fig. 6). The greatest reptilian contributions (>90% of all tetrapod HIPE) occur across the Middle East and North Africa (Fig. 4a). The lowest non-zero contributions of reptiles (<10%) occur across northern North America and Europe, the Andes and the Himalayas, where reptiles are scarce.

Globally, reptilian HIPE is only moderately correlated with HIPE patterns for other tetrapod groups in each cell, and inter-correlations are moderate between all groups (Supplementary Fig. 7). Turtle HIPE is consistently weakly correlated with that of other reptilian orders and tetrapod groups (spatially corrected correlation: *r* < 0.25, Supplementary Fig. 7). Global patterns of tetrapod HIPE are broadly congruent with those for reptiles but place further emphasis on the Atlantic coast of Brazil, the Caribbean, Central Africa and New Guinea (Fig. 4b). The variation in patterns of clade-specific contributions to global tetrapod HIPE (Fig. 4a, Supplementary Fig. 7) further highlights the importance of including all major tetrapod groups in analyses designed to represent the entire clade.

### Species prioritisation metric for conserving PD

PD naturally increases with species richness. However, when we sum the phylogenetic branch lengths for all species in each tetrapod group to estimate total PD, amphibians have the greatest PD of all tetrapod groups (median across the distribution of 100 phylogenetic trees = 130 BY for 7,239 species [93% of described species]) despite comprising fewer species than lepidosaurs (128 BY for 9,557 species [91%]) and birds (85 BY for 9,993 species [91%]). Together, turtles (8.3 BY for 293 species [81%]), crocodilians (0.5 BY for 23 species [96%]) and lepidosaurs comprise 137 BY of reptilian PD across 91% of reptile species. Finally, the 4751 (84%) mammal species in our analyses represent just 47 BY of unique PD (Supplementary Table 2). The distribution of PD values remained similar when we removed species at random from each phylogenetic tree to generate trees with equal proportions of species coverage for all clades (see Methods, Supplementary Table 3).

At the species-level, turtles contribute the largest amounts of unique PD per species, with the greatest median TBL (Fig. 1) of any tetrapod group (14.1 MY; compared to 8.5 MY for amphibians, 8.3 MY for crocodilians, 4.9 MY for lepidosaurs, 3.9 MY for mammals and 3.1 MY for birds), and lepidosaurs have the greatest unique contribution to PD by a single species (median TBL = 238.7 MY—*Sphenodon punctatus*). As previously reported^45^, the calculation of species-level measures of PD is largely robust to the influence of missing species and our results remain largely unchanged when repeated on our rarefied phylogenetic trees (Supplementary Table 3).

To identify species on long terminal branches responsible for large amounts of irreplaceable and range-restricted PD, we calculated what we term TE for all tetrapods for which spatial and phylogenetic data were available (~84% of species, Supplementary Table 2). TE is the terminal branch length (TBL) of a species multiplied by the reciprocal of its range size and is a fundamental component of the spatial PE metric (Fig. 1). Despite having shorter terminal branches than turtles, amphibians have the greatest median TE score across tetrapod clades (2.1 ×10^−4^ MY^−1^ km^2^), significantly greater than that of lepidosaurs (0.41 × 10^−4^ MY^−1^ km^2^), turtles (0.09 ×10^−4^ MY^−1^ km^2^), mammals (0.04 ×10^−4^ MY^−1^ km^2^), birds (0.01 ×10^−4^ MY^−1^ km^2^), and crocodiles (ANOVA: 0.007 ×10^−4^ MY^−1^ km^2^; adjusted *p*-values from Tukey HSD < 0.0001)—likely because of their small ranges.

To incorporate a measure of vulnerability with which to weight TE scores for conservation prioritisation, we developed a species-level counterpart to our spatial metric. HITE is an extension of TE that multiplies the terminal branch of a species by the reciprocal of the total human pressure-weighted score across its distribution (Fig. 1, Supplementary Table 1). Species that are endemic to a single grid cell under very high human pressure have a much lower human pressure-weighted distribution (and therefore greater reciprocal and greater HITE scores) than a widespread species occurring in regions of low pressure and thus a greater HITE score. Thus, high HITE scores highlight species that represent large amounts of unique PD, are endemic to regions of high human pressure, and thus likely in need of conservation attention.

**Fig. 5:**
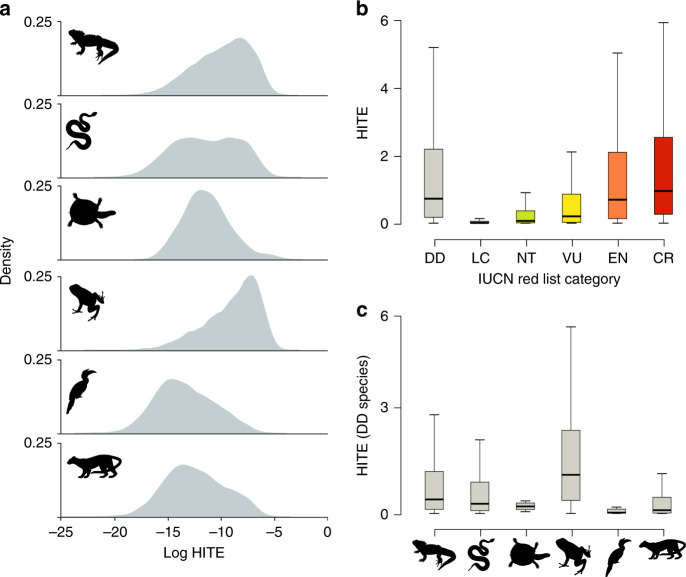
Distributions of human-impacted terminal endemism (HITE) for tetrapods. **a** Density distributions of log-transformed HITE scores for tetrapods. Species with long terminal branches occurring in very few grid cells under high human pressure score highly and fall on the right of the x-axis, whereas species with short terminal branches and large ranges encompassing regions of low human pressure fall on the left of the x-axis. Y-axis indicates density of species in each clade with a given HITE value. **b** Distribution of HITE scores (in 10^−3^ MY^−1^ km^2^) across tetrapods for each IUCN Red List category (excluding Extinct, Extinct in the Wild and unassessed species): Data Deficient (DD; *n* = 2604 spp., grey box); Least Concern (LC; *n* = 15217 spp., dark green box); Near Threatened (NT; *n* = 1709 spp., light green box); Vulnerable (VU; *n* = 2007 spp., yellow box); Endangered (EN; *n* = 1965 spp., orange box); Critically Endangered (CR; *n* = 1035 spp., red box). **c** Distribution of HITE scores (in 10^−3^ MY^−1^ km^2^) for Data Deficient (DD) tetrapod species for key tetrapod groups, from left to right on *x*-axis: lizards (*n* = 478 DD spp.), snakes (*n* = 404), turtles (*n* = 8), amphibians (*n* = 1222), birds (*n* = 35) and mammals (*n* = 457). For boxplots, centre line = median; box limits = upper and lower quartiles; whiskers = 1.5x interquartile range. Source data are provided as a Source Data file.

As with TE values, amphibians have the highest HITE values (median = 5.1 ×10^−4^ MY^−1^ km^2^; Fig. 5a), followed by lepidosaurs (9.9 × 10^−5^ MY^−1^ km^2^; lizards = 1.7 ×10^−4^ MY^−1^ km^2^, snakes = 2.8 ×10^−5^ MY^−1^ km^2^mammals (8.5 ×10^−5^ MY^−1^ km^2^), birds (3.0 ×10^−5^ MY^−1^ km^2^), turtles (2.5 ×10^−5^ MY^−1^ km^2^) and crocodilians (0.2 ×10^−5^ MY^−1^ km^2^).

### Data deficiency and PD loss

The omission of species lacking extinction risk data from estimations of PD loss can lead to considerable underestimations of potential loss of biodiversity^46^. Indeed, we find that Data Deficient tetrapods have significantly longer terminal branches (median = 5.4 MY) than those listed as Least Concern (4.3 MY), Near Threatened (4.3 MY) and Vulnerable (4.8 MY; adjusted p-values from ANOVA and Tukey HSD test < 0.001) and similar to Endangered (5.2 MY) and Critically Endangered (5.5 MY) species (adjusted *p*-values > 0.05).

Similarly, HITE scores of Data Deficient tetrapods (median = 7.2 ×10^−4^ MY^−1^ km^2^) are higher than those of Least Concern (6.3 ×10^−6^ MY^−1^ km^2^), Near Threatened (6.7 ×10^−5^ MY^−1^ km^2^) and Vulnerable species (2.0 ×10^−4^ MY^−1^ km^2^; adjusted p-values from ANOVA and Tukey HSD < 0.001), and are comparable to those of Endangered (6.9 ×10^−4^ MY^−1^ km^2^) and Critically Endangered species (9.5 ×10^−4^ MY^−1^ km^2^; adjusted p-values from ANOVA and Tukey HSD > 0.05; Fig. 5b). This indicates that Data Deficient species are similarly irreplaceable, have similarly small ranges, and are under comparable levels of human pressure as threatened species. Within Data Deficient species, amphibians have the highest HITE scores (median = 1.5 ×10^−3^ MY^−1^ km^2^), followed by lepidosaurs (4.7 ×10^−4^ MY^−1^ km^2^; lizards = 5.5 ×10^−4^ MY^−1^ km^2^, snakes = 3.8 ×10^−4^ MY^−1^ km^2^; Fig. 5c).

We ranked all tetrapod groups by their HITE scores to identify priority species that are irreplaceable and restricted to regions under high human pressure (Supplementary Table 4). Worryingly, four of the ten highest-ranking lizards and eight of the top ten snakes are listed as Data Deficient by the IUCN (ten highest-ranking HITE species for each clade in Supplementary Table 4). These 12 Data Deficient reptiles alone embody more than 500 million years of unique PD.

To explore the extent to which extinction threatens to erode the phylogenetic tree of life, we estimated the amount PD to be lost should all currently threatened species (Vulnerable, Endangered or Critically Endangered) become extinct (a worst-case scenario). If we lost all currently threatened crocodilians (11 species with phylogenetic data), we stand to lose 193 MY (~37% of crocodilian PD). The loss of all threatened turtles (134 species with phylogenetic data) would lead to a loss of 3.4 BY (~41% of turtle PD). The loss of all 1151 threatened lepidosaurs with phylogenetic data would result in the loss of around 9.5 BY (median, 8% of lepidosaur PD). Combined, we stand to lose more than 13.1 billion years (median; range = 12.3–14.3 BY), or around 10% of total reptile PD. This is 1.36 billion years more PD than if extinctions were randomly distributed within each reptilian order (paired t-test; *t* = 20.32, d.f. = 99, *p* < 0.0001). The potential loss of PD across threatened reptiles is significantly lower than that for amphibians, which stand to lose around 21 BY of PD (~16% of total PD). Birds stand to lose 6.2 BY (~7% of total PD) and mammals 6.4 BY (~13% of PD). Together, close to 50 billion years of unique tetrapod PD is at risk of extinction (~11% of total PD).

Given the large proportion of Data Deficient and unassessed reptiles (~10% and ~34% of all species, respectively), and their potentially high extinction risk, such loss of PD may be much greater, especially where data deficiency for both extinction risk and phylogenetic relationships intersect. For example, the enigmatic lizard genus *Dibamus* is represented by 22 species in our study (of the 24 species recognised globally^27^), sixteen of which are either unassessed or listed as Data Deficient by the IUCN (as of December 2019). Fifteen of these 22 species are included in the phylogeny we used despite having no genetic data available^47^, and 12 are known only from their type locality^48^.

The amount of PD represented by the 15 *Dibamus* species included in the phylogeny that lack genetic data is highly uncertain, and ranges from 260 to 1340 MY across 100 phylogenies (median = 560 MY). Accordingly, estimates of the amount of PD loss due to extinction of unassessed or Data Deficient species range across five orders of magnitude, from 0.1 MY (a single species lost with the shortest TBL across 100 phylogenies—0.00001% additional PD loss) to 1010 MY (all 16 unassessed/Data Deficient species lost with maximum branch lengths from 100 phylogenies—7.8% additional PD loss), with a median loss of 230 MY (1.8% additional PD loss).

## Discussion

Globally, reptiles (excluding avian reptiles) comprise significantly more PD than any other tetrapod group. The distribution of reptilian PD largely reflects global richness patterns^32^, though our analysis suggests that extremely high richness in snakes and lizards is achieved through shallow diversification within clades (Fig. 2). Our results highlight a large overlap between regions of high human impact and irreplaceable reptilian PD, which is much greater than expected if reptilian PD were distributed randomly with respect to human impacts. We therefore incorporated human pressure data into our spatial and species-level analyses to capture its potential impact on globally significant concentrations of range-restricted PD. Our metrics represent the first integration of data on environmental pressure affecting terrestrial vertebrates into global prioritisations of imperiled PD.

Reptiles have the highest scores of our spatial metric, HIPE, meaning they are faring worse than amphibians, birds and mammals, and contribute the highest levels of imperiled PD per grid cell. Reptilian contributions to global patterns of tetrapod HIPE are greatest in arid and semi-arid regions, particularly in the Middle East and Southern, Northern and the Horn of Africa (Figs. 3b, 4a)—areas often overlooked in global prioritisations of terrestrial conservation importance for other tetrapod taxa^10,22,23,33,36^. Thus, the inclusion of reptiles in global analyses of this kind is crucial to accurately value terrestrial vertebrate diversity for conservation at national, regional and global scales.

Global patterns of reptilian HIPE emphasise the importance of regions where large amounts of PD are under very high human impact; these are particularly pronounced across biodiversity hotspots^49^ in the Caribbean, the Western Ghats, the Philippines and Japan (Fig. 3c). These grid cells represent areas of high urgency for the conservation of global reptilian PD and would perhaps benefit most from restorative conservation actions. Conversely, regions where PD is largely restricted to regions of low human pressure would benefit most from the establishment, enforcement and expansion of protected areas to safeguard irreplaceable and not-yet-impacted diversity (Supplementary Fig. 4).

It is important, however, to acknowledge the coarse scale of our analyses when highlighting grid cells of high conservation value and when directing targeted conservation actions for effectively capturing imperiled and irreplaceable diversity on the ground. This is particularly crucial in regions under high human impact, where aggregations of highly range-restricted species, which drive patterns of high HIPE, are likely confined to extremely small pockets of remaining suitable habitat within our coarse-resolution grid cells.

Indeed, our coarse spatial resolution can potentially mask more intricate patterns of species distributions and remaining habitat at finer resolutions. However, Venter et al.^42^ uncovered similar relationships between regions of high human pressure, biodiversity hotspots and threatened species distributions at a fine spatial scale. Further, the distribution of fine-resolution Human Footprint data comprising our coarse-resolution analyses indicates regions under very high human pressure have few, if any, low pressure fine-resolution grid cells remaining (Supplementary Fig. 8).

Our categorisation of the original Human Footprint values into the five broader categories of human pressure defined by Venter et al.^42^ may affect our results where they involve grid cells whose untransformed Human Footprint values fall close to the border between adjacent categories of human pressure. However, there is no single accepted method for incorporating Human Footprint data into global analyses of biodiversity conservation^42,50,51^, and the differences between those that do exist are subtle and unlikely to affect our main conclusions. Nevertheless, further work is needed to determine how best to interpret Human Footprint data, and we designed our HIPE and HITE methods with the modularity to enable future applications of these metrics to benefit from improved weightings should these become available.

At the species level, reptiles embody more unique evolutionary history than amphibians, birds or mammals. Turtles have particularly long terminal branches, indicating that each turtle species, on average, represents large amounts of unique evolutionary history. It is troubling to note that, across tetrapods, Data Deficient and threatened species also generally comprise more unique evolutionary history than non-threatened species.

Our species-level metric, HITE, prioritises species with long terminal branches restricted to small ranges under high human impact. Many small-ranged amphibians and lizards tend to be on long terminal branches and occur in areas of high human impact. Our metric, HITE, highlights these groups as of major conservation concern.

Many of the highest-ranking HITE tetrapods which have also been classified by the IUCN Red List as Endangered or Critically Endangered are also recognised as priority Evolutionarily Distinct and Globally Endangered (EDGE) species^52^. However, as HITE does not consider IUCN Red List extinction risk data, and uses only phylogeny, range size, and human pressure, we also identify species of conservation importance which are currently unassessed or listed as Data Deficient or non-threatened by the IUCN Red List. Indeed, we found that Data Deficient tetrapods tend to have HITE scores comparable to those of species listed as Endangered or Critically Endangered.

This pattern is particularly pronounced in squamates and amphibians, where considerably greater proportions of the highest-ranking HITE species for these groups are Data Deficient than either birds or mammals. This suggests that many of the poorly known amphibians and reptiles are likely to be highly evolutionarily distinct and restricted to regions of intense human pressure. Although such prevalence of high-ranking Data Deficient HITE species is likely driven by higher proportions of data deficiency in amphibians (22%) and reptiles (15%) compared with mammals (14%) and, especially, birds (0.5%)^29^, it also highlights that many species in areas of high human impact may well be at high risk of extinction.

Our case study of the poorly known lizard genus *Dibamus* underlines the amount of uncertainty we currently face when identifying conservation priorities and estimating impacts of species loss across the tree of life. Our estimation of potential loss of PD in this clade varies across four orders of magnitude depending on our assumptions of uncertainty in both phylogeny and extinction risk. Although this is an extreme example, our lack of knowledge of extinction risk and phylogenetic relationships across large parts of the tree of life means any estimations of potential biodiversity loss may be significant underestimates. Improved efforts to address these data deficiencies are critical for both accurate estimations of global trends in diversity loss and for informing applied conservation action. Improved ecological and distribution data for poorly known species would permit fine-scale analyses of the impacts of human activities on the extent of suitable habitats available for species at a global scale.

Without conservation action we face the loss of billions of years of unique amphibian and reptilian evolutionary history worldwide. While greater research efforts are needed to elucidate the phylogenetic relationships, distribution and population status of poorly known reptiles and amphibians, current and future conservation efforts also need to focus on regions, lineages and species that comprise disproportionate amounts of irreplaceable and imperiled PD.

## Methods

### Data

We used updated distribution polygons for turtles, crocodilians and lepidosaurs from the Global Assessment of Reptile Distributions (GARD)^32^. We used published phylogenies for lepidosaurs^47^, crocodilians^53^ and turtles^54^. The crocodilian and turtle phylogenies used were single, consensus, fully resolved phylogenies. To capture phylogenetic uncertainty around the taxonomically imputed lepidosaur phylogenies, we randomly sampled 100 fully resolved phylogenies from a distribution of 10,000 trees^47^ and used each phylogeny in our analyses to generate median values of PD and PD-based metrics. We matched the species in each phylogeny to the distribution data using the taxonomy from the July 2018 version of the Reptile Database^27^.

For our spatial analyses we included only species with both phylogenetic and distribution data (9,862 species or 91% of total reptilian diversity; Supplementary Table 2). Each species distribution polygon was converted to grid cells in a Mollweide equal area projection at 96.5 × 96.5 km (~1 degree at the equator) resolution^32^. Analyses of this nature are typically conducted at such a coarse resolution to reduce false absences in the distribution data and improve accuracy at the cost of precision for spatial results^5,10,23,32,55^. This approach leads to the overestimation of range size of species with extremely narrow distributions, particularly if they overlap with the borders of multiple grid cells. It considers them equivalent in range size to species with larger distributions that are still captured within the same number of 96.5 km resolution grid cells. This may potentially reduce the influence of these narrowly distributed species on our results. However, previous analyses of reptile spatial patterns at the global scale which weighed richness by range size have found results to be qualitatively unchanged when conducted at a finer resolution^32^.

We conducted our spatial and species-level analyses for crocodilians, lepidosaurs and turtles separately and also considered the three orders together as ‘reptiles’ with the exclusion of birds (Aves), which are nested within the Reptilia. We also conducted our analyses across the other tetrapod classes—amphibians, birds and mammals—for comparison with reptiles. We extracted a random sample of 100 phylogenetic trees from published phylogenies for amphibians^56^, birds^22^ and mammals^57^ and spatial data, as polygon shapefiles, for amphibians and mammals from IUCN^29^ and for birds from BirdLife International^58^. These distribution data were subset to contain only native and resident or breeding ranges. As with reptiles, for our spatial analyses we included only species with both phylogenetic and distribution data (5786 amphibians (75.5% of species); 9274 birds (84.5%); 4386 mammals (77%)—this is ~84% of all tetrapods, including reptiles; Supplementary Table 2) and calculated median values of PD and PD-based metrics for each grid cell.

We used the 2009 Human Footprint index (HF)^42^—the most up-to-date HF dataset—to designate spatial patterns of human pressure. The HF index evaluates each grid cell based on the intensity of eight measures of human pressure (built environments, crop land, pasture land, human population density, night-time lights, railways, roads, navigable waterways), weighted according to estimates of their relative levels of human pressure^41,42^, and assigns an HF value between 0 (lowest human pressure) and 50 (greatest human pressure) to each cell^42^. We up-scaled the HF data from its original 1 × 1 km resolution to our 96.5 × 96.5 km grid for species distribution data by taking the mean value from all 1 km x 1 km grid cells coincident with each 96.5 km x 96.5 km grid cell (Supplementary Fig. 8).

### Spatial value metric for conserving PD

As small range size is linked to elevated extinction risk^39,59^, if small-ranged species are clumped together on the tree of life, with no shared branches also subtended by a wide-ranging species, a disproportionately large amount of PD may be at risk of extinction. To examine whether small range size is phylogenetically conserved in this manner, we calculated Pagel’s lambda^60^ for crocodilians, turtles, and lepidosaurs separately and—within lepidosaurs—for lizards and for snakes independently, to remove the bias caused by large range sizes of snakes from the analysis of lizard distributions^32^. Pagel’s lambda provides an estimate of how phylogenetically conserved a trait is across a phylogeny, with scores close to 1 indicating a trait is strongly constrained on the phylogeny, whereas scores close to 0 indicate a trait to be randomly dispersed throughout the phylogeny^60^.

To map global patterns of reptilian PD, for each grid cell occupied by more than one species, we summed the lengths of all branches between the root node and tips for the subtree comprising all species in the grid cell. When only one species was present in a grid cell the length of the terminal branch (the branch connecting the species ‘tip’ to the rest of the phylogeny) was used to represent PD. As the branch lengths are time-calibrated, the resulting values represent the PD, as units of time, present in each grid cell.

We summed the branch lengths of the turtle and crocodilian phylogenies, and combined these with the median summed branch lengths from the 100 lepidosaur phylogenies to estimate total global reptilian PD. As reptiles are paraphyletic with regards to birds, any estimations of reptilian PD for grid cells containing at least one lepidosaur and one of either turtles or crocodilians are conservative as they omit the lengths of the phylogenetic branches connecting the three lineages within reptiles. Though crocodilians were included in analyses of all reptiles, we do not report their individual results because they comprise of only 25 species^27^.

We explored the relationship between PD and richness for each reptile group using Pearson’s correlation corrected for spatial autocorrelation in the R package ‘Spatialpack’^61,62^, with a conservative Bonferroni correction for multiple testing. To identify global variation in the relationship between PD and richness, we then calculated the residuals from a linear regression of richness against PD for all grid cells. We consider grid cells harbouring more PD than expected for the observed richness to represent regions of disproportionately phylogenetically diverse species compositions.

It is common to measure the deviation of PD from expected given the observed species richness using randomisation methods^23,33,63^, as the variance of the residuals around a regression decreases as the species richness of a grid approaches the number of species in the phylogeny^64^. However, the maximum grid cell richness for lepidosaurs never reaches 2% of the number of species in the phylogeny in our analyses (max grid cell richness = 161, with 9557 species in the phylogeny) nor 10% for turtles (max grid cell richness = 22, with 280 species in the phylogeny). Concurrently our model residual variance is independent of cell richness (Supplementary Fig. 9). On the other hand, using null models to estimate deviation from expectation when applied to our data resulted in extreme decreases in variance as richness increased, leading to only regions of very low species richness to exhibit deviation from expectation (Supplementary Fig. 9). We therefore consider the use of residuals as valid in our case of extreme low grid cell species richness relative to number of species in the phylogeny.

For later comparison with our own PD-based spatial metric, we calculated three additional metrics: the species-based metric Weighted Endemism (WE), which provides a measure of range-size-weighted species richness^8,32^, and two PD-based extensions of Weighted Endemism: EDR^22^ and PE^8^ (Supplementary Table 1). Whereas mapping PD requires the summing the PD of all branches occurring in each grid cell, meaning the PD of branches is counted multiple times, EDR and PE share PD equally amongst grid cells so that, when summed across all grid cells in an analysis, total EDR or PE are equal to the total PD of all branches mapped. EDR achieves this by first sharing the PD of the tree amongst all species using the ‘fair proportion’ approach^65^, which divides the PD of each branch equally amongst each descendant species. This Evolutionary Distinctiveness^6^ score is then shared equally across the grid cells in which the species occurs^22^. PE, on the other hand, divides the PD of each branch equally across all grid cells in which any species descendant to the branch occur^8^.

A key difference between EDR and PE is in their treatment of species ranges: EDR treats all species ranges as spatially independent whereas PE accounts for the spatial overlap of species. We suggest that EDR and PE therefore better represent the potential loss due to differing drivers. EDR represents the amount of Evolutionary Distinctiveness imperiled by species-specific threats (e.g. targeted hunting); the losses are species focused because only range size (and not range overlap with other species) is accounted for. In contrast, PE represents the amount of PD attributed to a particular unit of space, reflecting the impact of landscape-level threats (e.g. habitat loss); having additional descendent species in the same size region makes no difference to extinction risk of phylogenetic branches because loss of the region would impact all those species together. As most threats to tetrapod species are present at the landscape-level (e.g. agriculture, logging and livestock production)^66–68^, we hereafter report and develop analyses based on the PE metric and later develop TE to circumvent the differences between PE and EDR.

As biodiversity hotspots and concentrations of threatened amphibians, birds and mammals coincide with regions under high human pressure^42^, we explored the relationship between concentrations of irreplaceable reptilian diversity and human pressure using a spatially corrected Pearson correlation of reptile PE and Human Footprint values between 0 and 50 across all grid cells containing at least one reptile species.

The relationship between Human Footprint scores and the impact of human activities on biodiversity is not linear: a Human Footprint score of just 4 (out of 50) is equivalent to pasture, or “human dominated” landscapes^69^, a score of 7 is equivalent to agriculture and scores above this represent highly modified landscapes^50^. This means the difference between grid cells with Human Footprint scores of 0 (pristine) and 4 (pasture) is likely much greater than the difference between grid cells with Human Footprint scores of 46 and 50.

To account for this, we elected to use pre-defined categories of human pressure derived from these general stratifications in our global analyses. One option was to develop a novel categorisation of the data derived from Allan et al.’s^50^ stratification (e.g. Human Footprint scores lower than 3 = lowest human pressure, a score of 3 = low pressure, 4 = ‘pasture’ or equivalent, 5–6 = high pressure, 7 = agriculture or equivalent, and 8–50 = highly modified landscapes). However, rather than establishing a new categorisation scheme for our analyses we adopted that of Venter et al.^42^ who used biologically relevant stratifications to partition 51 levels of Human Footprint (0–50) into five broad categories of human pressure, for use in global analyses incorporating biodiversity data. Each of these categories is defined as: “no pressure” (HF = 0), “low pressure” (HF = 1–2), “moderate pressure” (HF = 3–5), “high pressure” (HF = 6–11), and “very high pressure” (HF ≥ 12)^42^, and represents approximately 20% of the earth’s terrestrial surface.

Any binning of the original HF data into broader categories has the potential to amplify relatively small differences in the raw data (e.g. under our categorisation method, a grid cell of HF = 11 and one of HF = 12 are considered less similar than they are to grid cells where HF = 6 and HF = 50, respectively), leading to potential under- and over-estimations of human pressure across grid cells, relative to one another. However, this problem is inherent in any categorisation of human pressure data—from the original 51 HF categories (0–50) at one extreme to a binary categorisation at the other—due to the non-linear, and currently poorly understood, relationship between HF and its impact on biodiversity.

We adopted these five categories of human pressure of Venter et al.^42^ to determine whether there were greater levels of reptile PE in regions under higher human pressure using ANOVA and Tukey’s Honest Significant Differences Test. The use the five categories of human pressure outlined by Venter et al.^42^ rather than the 51 fine-scale values for Human Footprint also improves the accuracy (at the expense of precision) of grid cell value assignment when upscaling the spatial data from 1 km x 1 km to 96.5 ×96.5 km resolution to match the species distribution data (Supplementary Fig. 8).

To test whether regions of high PE are coincident with high human pressure at greater levels than we would expect if human pressure was distributed randomly across the global distributions of reptiles, we followed Venter et al.^42^ by selecting the richest 10% of grid cells for reptilian PE (hereafter ‘high value grid cells’) and calculated the proportion of these high value grid cells that are also deemed to be under high or very high human pressure (Human Footprint ≥ 6)^42^. We then redistributed observed Human Footprint values at random across all terrestrial grid cells in which reptiles occur and recalculated the proportion of high value grid cells now considered to be under high or very high human pressure. We repeated this randomisation 1000 times to generate a distribution of randomised overlap scores for comparison with the observed proportion of overlap.

Whilst PE incorporates the intrinsic threat of small range size into the calculation of grid cells for conservation of unique evolutionary history, it does not measure the myriad extrinsic threats present. We therefore incorporated Human Footprint (HF) data^41,42^, in the form of Venter et al.’s^42^ five categories of human pressure (HP), as a measure of vulnerability.

To calculate the human pressure (HP)-weighted spatial distribution of a phylogenetic branch we first linearly scored each terrestrial grid cell in the distribution according to which of the five classes of human pressure the grid cell belongs. These are: 1 = ‘no pressure’ (Human Footprint = 0); 0.8 = ‘low pressure’ (HF = 1–2); 0.6 = ‘moderate pressure’ (HF = 3–5); 0.4 = ‘high pressure’ (HF = 6–11); 0.2 = ‘very high pressure’ (HF = 12–50)^42^ (see Supplementary Fig. 8). These weightings are not intended to account for finer scale gaps in the range (spatial distribution) due to human pressure, but rather to provide a better proxy for threat than range size alone. As the Human Footprint scores, and subsequent human pressure categories, are the result of an ensemble of threats that vary in nature, the true proportion of remaining suitable habitat will differ across grid cells of equal human pressure (Supplementary Fig. 8) and will also be species and disturbance-specific. Our scoring of grid cells based on broad categories of human pressure provides a relative weighting under the assumption that increased human pressure in a cell will have a negative impact on all coincident species^42^.

The new ‘HP-weighted distribution’ of a species is given by the sum of HP-weighted grid cell scores for all cells across which a species is distributed. We purposefully do not clip the ranges of species to remove regions of high human pressure, as in other analyses^70,71^. Our reasons for this are twofold. First, we consider regions under high human pressure to be of conservation importance, particularly when coincident with significant levels of endemic diversity, and did not want to exclude them from our analyses. Second, the fine-scale habitat association and environmental data required to accurately conduct such analyses are lacking for most terrestrial vertebrates at this time.

We used the HP-weighted distributions to calculate a new spatial PD metric, derived from PE, which we term HIPE. This approach apportions the PD of each branch of the phylogeny according to each grid cell’s contribution to the total HP-weighted distribution of the branch (Fig. 1, Supplementary Table 1). When a branch is found either in one grid cell or in multiple grid cells of the same HP-weighted grid cell value, HIPE is equivalent to PE in apportioning PD. However, when a branch occurs in grid cells that differ in their HP (i.e. that vary in human pressure), PD is apportioned by the relative contribution of the HP-weighted grid cells. Thus, grid cells with lower human pressure (higher HP-weighted grid cell score) receive a greater proportion of PD to reflect their higher present value. Consequently, branches that are entirely distributed across grid cells of high human pressure contribute a greater proportion of PD to highly impacted grid cells than branches that also occur in grid cells under low human pressure.

Consider a grid cell under high human pressure (HP-weighted score = 0.2) where only two branches are present, both comprising 10 MY of PD. Both branches also occur in one other grid cell, branch A in a low impact grid cell with a HP-weighted score of 1 (for a total HP-weighted distribution score of 1.2) and branch B in a high impact grid cell with a HP-weighted score of 0.2 (total HP-weighted range of 0.4, or two grid cells scored at 0.2). Under traditional PE, the grid cell receives 50% of the PD from each branch (5 MY) as it comprises 50% of the total distribution of the branch (one of two grid cells). Under HIPE, however, branch A would apportion only 1/6th (1.667 MY) of its PD to the grid cell as it contributes only 1/6th of the total HP-weighted distribution score (0.2 of total 1.2), with the remaining 5/6th of the PD being apportioned to the grid cell with a HP-weighted score of 1. Conversely, as branch B occurs only in two grid cells of HP-weighted score 0.2, the grid cell comprises 50% of the HP-weighted distribution of the species (0.2 of total 0.4) and is apportioned 50% of the PD of the branch (5 MY) (Supplementary Table 1; Fig. 1).

The PE metric simply distributes the PD from phylogenetic branches equally across all grid cells in which they occur. We view the HIPE metric as a specific application of a more general ‘modular’ extension of PE where, rather than distributing the PD from branches equally across space, PD is distributed in relation to weightings derived from other spatially explicit data provided—in this case broad human pressure data. For example, spatially explicit data on abundance, climate change vulnerability, protected area coverage, or taxon-specific anthropogenic impacts could be used to identify regions of high value for conservation. While we did not perform any spatial prioritisation exercises here, such data could also be employed under a phylogenetic complementarity framework to effectively prioritise grid cells for conservation action under different scenarios^5,10^.

HIPE increases the relative importance of grid cells under low human pressure as well as capturing cells under high human pressure with highly endemic PD. It is therefore important for conservation planning to highlight which of the high value regions (based on HIPE) are driven by endemic PD in areas of high vs. low human pressure, as the two extremes are likely to require different conservation action. By calculating the ratio of HIPE/PE for each grid cell we get a measure of the level to which phylogenetic branches in the grid cell are entirely restricted to grid cells of the same human pressure. As outlined above, HIPE = PE for a grid cell when all phylogenetic branches in the grid cell are distributed amongst grid cells under the same human pressure. Therefore, we can map the distribution of this ratio (HIPE/PE) for grid cells under both extremes of human pressure.

As HIPE redistributes PD to regions of lower pressure, grid cells under very high human pressure cannot have a HIPE/PE ratio greater than 1 as they cannot receive additional PD from grid cells under greater human pressure. Conversely, grid cells under no human pressure cannot have a HIPE/PE ratio lower than 1, as they can only gain PD when it is redistributed based on human pressure. We therefore partitioned global patterns of reptilian HIPE into two components: (1) regions under very high human pressure (HF ≥ 12) where the HIPE/PE ratio approaches 1, indicating an overwhelming proportion of the PD found in those grid cells is restricted to regions under very high human pressure and does not also occur in regions under lower human pressure; and (2) regions under no human pressure (HF = 0) where the HIPE/PE ratio approaches 1, indicating the vast majority of PD present in those grid cells is restricted to regions under no human pressure.

We mapped HIPE for all reptile orders individually and for all reptiles combined. To determine the regions where reptiles provide the greatest contributions to global patterns of tetrapod HIPE, we also calculated HIPE for mammals, birds, amphibians and for tetrapods as a whole. We then calculated the proportions of observed HIPE for all tetrapods that were contributed by each tetrapod clade. We present HIPE scores in MY^−1^ km^2^, where the HP-weighted distribution represents the area across which the scores are divided (e.g. a 96.5 × 96.5 km grid cell with a HP-weighted grid cell score of 0.2 is considered to comprise 1/5th of the area of an entire grid cell).

As the proportions of total species with phylogenetic and spatial data available varies across tetrapod clades (i.e. amphibians are less well represented in the phylogeny than other clades; Supplementary Table 2), we repeated our calculations of global HIPE values using phylogenetic trees for each clade that had been rarefied at random to match the 75.5% of species completeness observed in our amphibian data. For birds, mammals and lepidosaurs, for which we had a distribution of phylogenetic trees, we randomly removed the required number of species to reach as close to 75.5% of total clade richness as possible once from each of the 100 phylogenetic trees and recalculated HIPE. For turtles and crocodilians, for which we had a single consensus phylogenetic tree, we randomly dropped the required number of species from the full phylogenetic tree 100 times to generate a 100-tree distribution of species compositions and recalculated HIPE.

As with earlier analyses of relationships between grid cells, we ran Pearson’s correlation corrected for spatial autocorrelation in the R package ‘Spatialpack’^61,62^, with a conservative Bonferroni correction for multiple testing, to examine relationships between HIPE, PE and EDR. This provides an estimate of the extent to which these measures capture the same global patterns. We also ran these spatially corrected correlations for relationships between global HIPE patterns among reptile groups and between reptiles and other tetrapods, all with Bonferroni correction.

### Species prioritisation metric for conserving PD

We estimated the total PD of reptiles by summing the branch lengths of the crocodilian and turtle phylogenies and adding these to the summed branch lengths for each of the 100 lepidosaur phylogenies to generate a distribution of 100 total reptilian PD values. We compared this distribution with that for other tetrapod groups, which we generated by summing the branch lengths of the 100 random phylogenies for amphibians, birds and mammals. To generate a species-level measure of unique PD across all species, we used the median TBL for each species and compared the distribution of TBL scores across tetrapod clades using ANOVA and Tukey’s Honest Significant Differences Test. Branch length data were extracted for all phylogenies prior to the removal of species with no spatial data to limit the impact of differing availability of spatial data across the different classes on our species-level analyses. Species-level PD metrics, which are predominantly composed of TBL scores of species, have been shown to be robust to the omission of species from the phylogeny^45^. However, to further control for differences in taxonomic completeness, we repeated these analyses using the rarefied trees outlined above for each clade and recalculated total PD and median TBL.

To identify species that should be prioritised to preserve unique evolutionary history, we isolated the only component common to both PE and EDR: TBL weighted by range size. We define this as terminal endemism (TE); the TBL of a species multiplied by the reciprocal of the number of grid cells occupied by the species. If a species is found in only one grid cell, then its loss from that grid cell would result in the loss of its entire terminal branch. The TE of a species is implicitly calculated when calculating both EDR and PE and represents the unique contribution of the species to the total for each metric. We posit that, as a species-focused measure, TE circumvents the differences between EDR and PE when handling internal branches and retains the most essential component of each.

To incorporate human pressure into our prioritisation of species for conservation, we developed an extension to our novel TE metric, ‘HITE’. This metric is given by the TBL of a species multiplied by the reciprocal of its human pressure-weighted distribution score (see above). For example, a species with a TBL of 10 MY that is found in two grid cells, with HP-weighted grid cell scores of 0.2 and 1 would receive a HITE score of 10*(1/(1 + 0.2)) = 8.34. Under standard TE the same species would receive a lower score of 5: (10*(1/2)). HITE therefore increases in response to terminal branches occurring in grid cells under high human impact.

We calculated the TE, HP-weighted distributions, and HITE for all tetrapods and ranked the species from each clade to identify the species with the highest HITE scores.

### Data deficiency and PD loss

We highlight tetrapod species which are either unassessed or listed as Data Deficient by the IUCN but have a high HITE score. These are species that, due to their high irreplaceability and extremely restricted and human-impacted range, are priorities for conservation assessment. We compared HITE scores for tetrapods across IUCN Red List categories, using ANOVA and Tukey’s HSD test, to determine the relationship between HITE scores, data deficiency, and extinction risk across reptiles and all tetrapods.

To estimate how much reptilian PD may be lost if all threatened species were to become extinct, we dropped all species listed in threatened categories on the IUCN Red List (i.e. Vulnerable, Endangered and Critically Endangered) from their respective phylogenies and calculated the reduction in total PD. For lepidosaurs we did this for all 100 phylogenies to generate a distribution of values. We repeated these analyses for amphibians, birds and mammals to estimate the amount of tetrapod PD at risk of extinction.

To determine whether the potential loss of PD was greater than if extinction risk was randomly distributed across the reptilian tree of life, we then selected 100 random sets of species corresponding to an equal number of species as those observed to be threatened and dropped them from their respective phylogenies. We then compared the distribution of potential PD loss from species observed to be threatened with the distribution generated from randomised extinction using a paired t-test.

As it is likely that a significant proportion of unassessed and Data Deficient species are also threatened with extinction^72,73^, these estimates of loss of PD are conservative. To explore how data deficiency affects potential losses of PD across data-poor regions of the tree of life, we selected a poorly known squamate genus as a case study. We estimated the amount of PD lost under different scenarios of phylogenetic relationships and extinction risk for *Dibamus*, one of the least-known reptilian genera (and the sister clade to all other squamates). First, we estimated the amount of PD represented by the *Dibamus* species included in the phylogeny despite lacking genetic data across our random selection of 100 lepidosaur phylogenies. Second, we estimated how much PD would be lost under three extinction scenarios for *Dibamus*: (1) only a single unassessed or Data Deficient species becomes extinct; (2) a random number and selection of unassessed or Data Deficient species become extinct; and (3) all unassessed and Data Deficient species become extinct.

### Reporting summary

Further information on research design is available in the Nature Research Reporting Summary linked to this article.

## Supplementary information

Supplementary Information

Reporting Summary

## Data Availability

The data that support the findings of this study are available from the corresponding author upon request. The source data underlying Fig. 5a–c are provided as a Source Data file.

## Source data

Source Data

## Code availability

The R code for calculating the novel metrics presented here are available on GitHub (https://github.com/rgumbs/HIPE) and from the corresponding author upon request.
