# Supplementary material for: Global priorities for conservation of reptilian phylogenetic diversity in the face of human impacts
Source: Nat Commun. 2020 May 26;11:2616. doi: 10.1038/s41467-020-16410-6 (PMC7250838; doi:10.1038/s41467-020-16410-6)
Supplement: Supplementary file 1 — Supplementary Information [file 41467_2020_16410_MOESM1_ESM.pdf]

Supplementary Information

Global priorities for conservation of reptilian phylogenetic diversity in the face of human impacts

Gumbs et al.

Contents

Supplementary Figure 1: Phylogenetic distribution of range size across reptiles.

Supplementary Figure 2: Global patterns of reptilian phylogenetic diversity (PD).

Supplementary Figure 3: Relationships of rarity-weighted spatial metrics for all reptiles.

Supplementary Figure 4: Regions under no human pressure with largely endemic reptilian phylogenetic diversity (PD).

Supplementary Figure 5: Global patterns of Human-Impacted Phylogenetic Endemism (HIPE) for reptilian clades.

Supplementary Figure 6: Global contributions to tetrapod Human-Impacted Phylogenetic Endemism (HIPE) by non-reptilian tetrapods.

Supplementary Figure 7: relationships amongst reptile and tetrapod groups for all grid cells of global Human-Impacted Phylogenetic Endemism (HIPE).

Supplementary Figure 8: Distribution of untransformed Human Footprint values when aggregated to lower resolution 'Human Pressure' grid cells.

Supplementary Figure 9: Variance of values for three methods to measure the deviation from expected phylogenetic diversity (PD).

Supplementary Table 1: Mathematical definitions of phylogenetic diversity and endemism-based metrics used in study.

Supplementary Table 2: Taxonomic representation for each reptilian order and tetrapod class in this study.

Supplementary Table 3: Median diversity metric scores for each clade under equal taxonomic

representation.

Supplementary Table 4: The ten highest ranking Human-Impacted Terminal Endemism (HITE) species for each tetrapod group.

Supplementary references

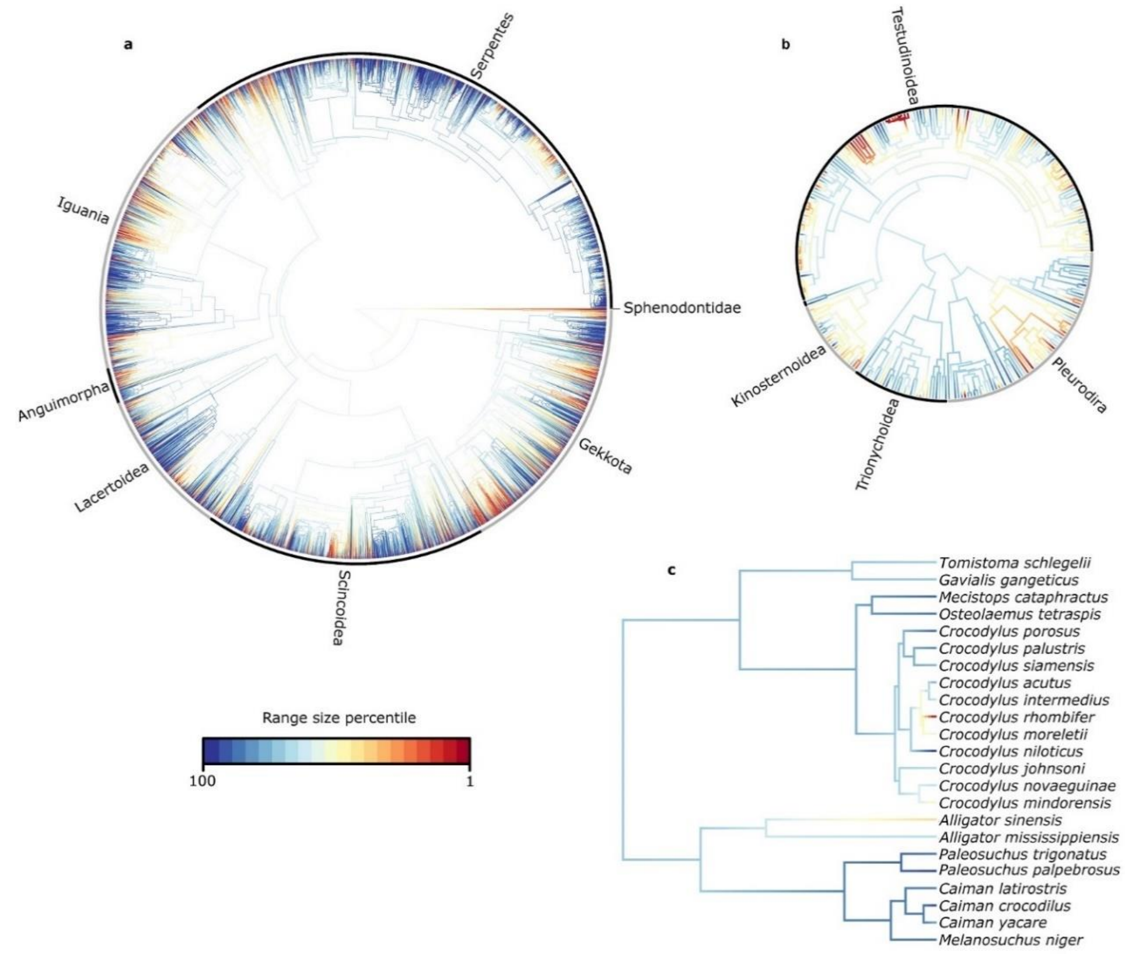

Supplementary Figure 1: Phylogenetic distribution of range size across reptiles. The percentiles of range size, measured in number of grid cells, across the phylogenies of a) lepidosaurs (squamates + tuatara), b) testudines, and c) crocodilians.

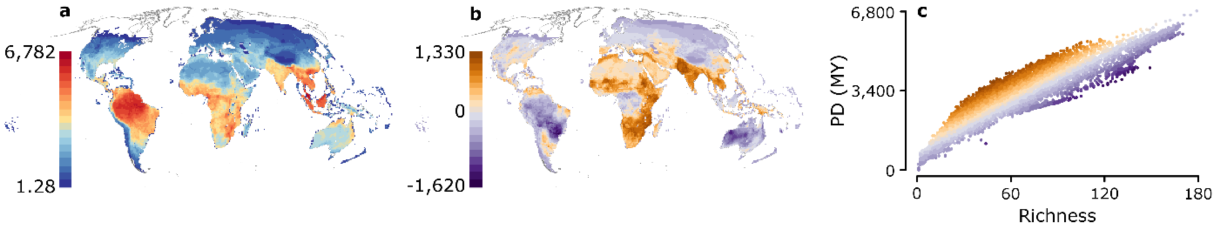

Supplementary Figure 2: Global patterns of reptilian phylogenetic diversity (PD). Cumulative PD (a); amount of PD per grid cell greater or lower than expected for the observed species richness (i.e. residuals from linear regression of the two variables) (b); and the relationship between richness and PD across all grid cells (c) for all reptiles. For panel c, colours correspond to residual grid cell values from panel b.

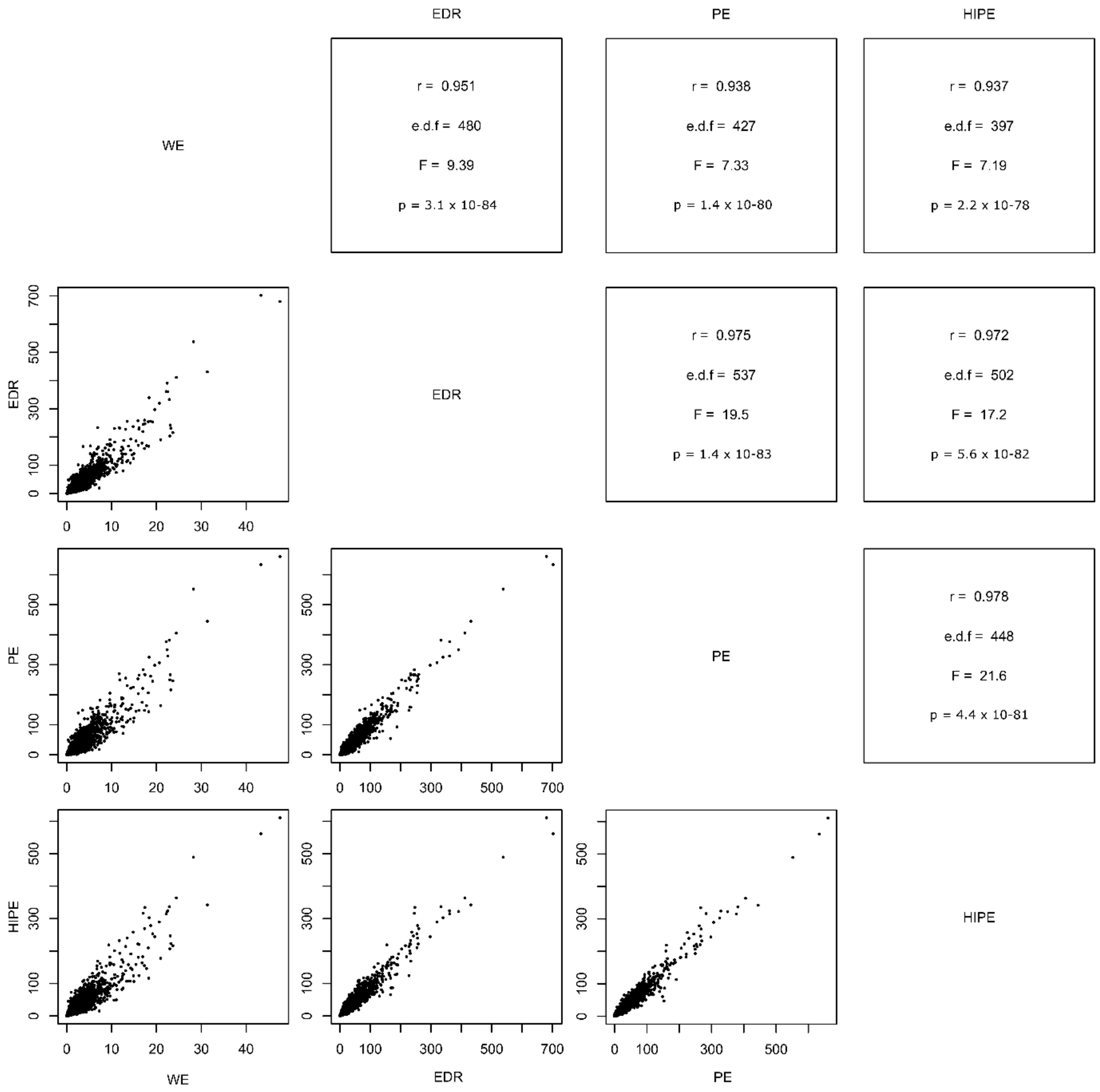

Supplementary Figure 3: Relationships of rarity-weighted spatial metrics for all reptiles. Results of spatially corrected Pearson's correlations among Weighted Endemism (WE), Evolutionary Distinctness Rarity (EDR), Phylogenetic Endemism (PE) and Human-Impacted Phylogenetic Endemism (HIPE) (above diagonal split) and scatterplots of the values for each grid cell of global reptile distribution for each metric (below diagonal split).

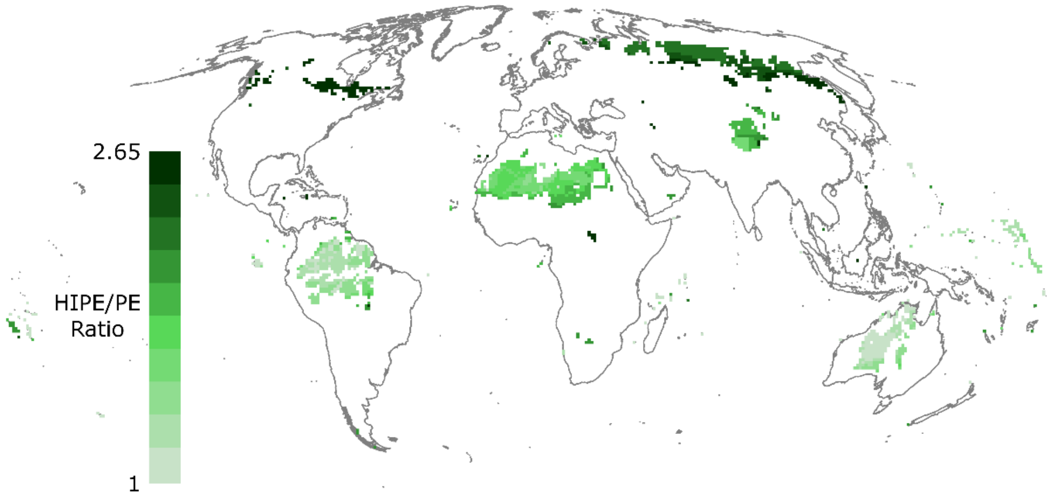

Supplementary Figure 4: Regions under no human pressure with largely endemic reptilian phylogenetic diversity (PD). The ratio of Human-Impacted Phylogenetic Endemism (HIPE) to Phylogenetic Endemism (PE) for grid cells under no human pressure (HP < 1). A ratio of 1:1 means all PD found in the grid cell is restricted to regions of no human pressure, and the higher this becomes, the greater the proportion of PD also distributed in regions under increasing human pressure. Darkest green rid cells have the greatest HIPE to PE ratio, and the lightest green have the lowest HIPE to PE ratio.

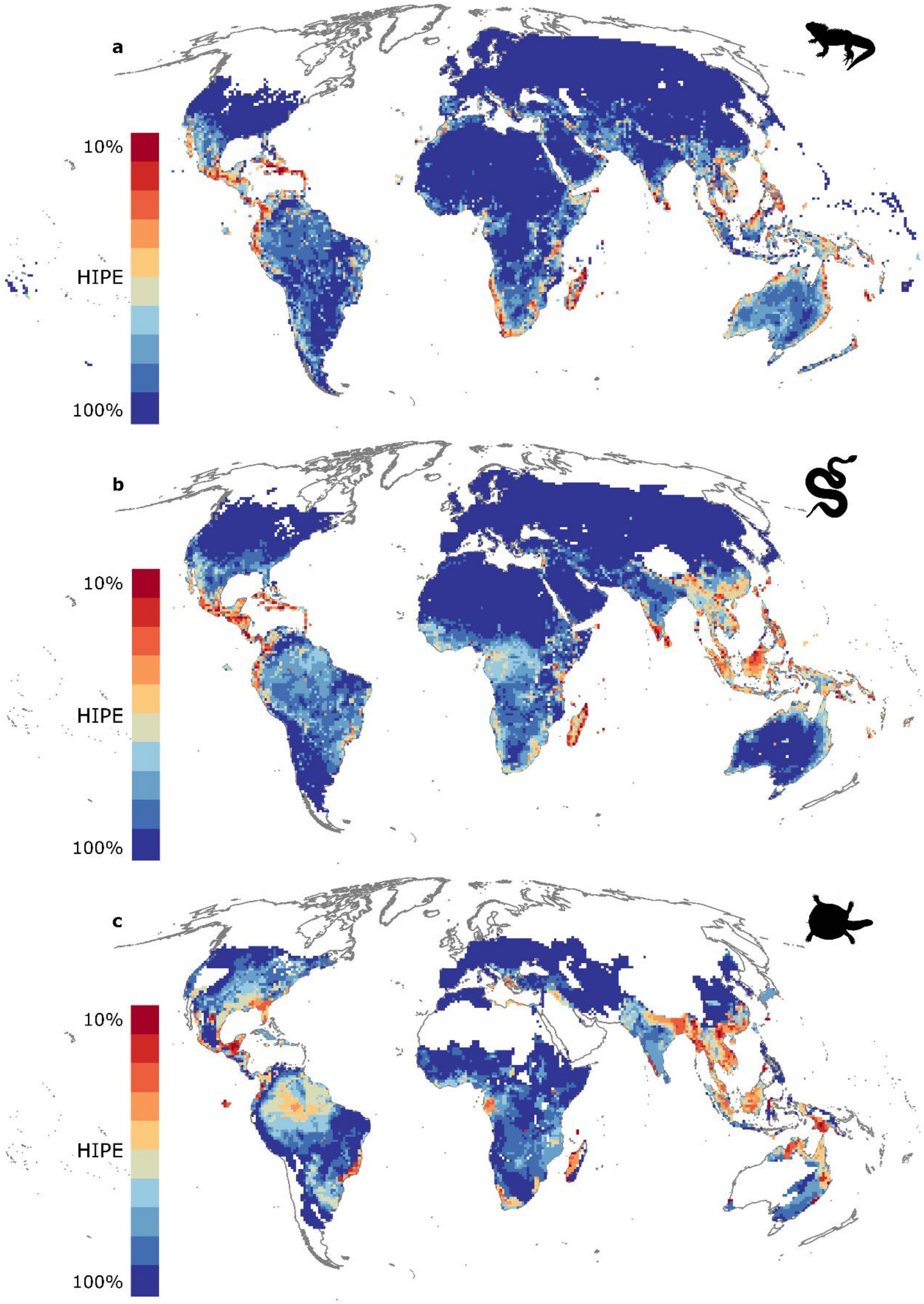

Supplementary Figure 5: Global patterns of Human-Impacted Phylogenetic Endemism (HIPE) for reptilian clades. The global patterns of HIPE for a) lizards (lizards, amphisbaenians and the tuatara), b) snakes and c) testudines. The top 10% ranked grid cells for HIPE are darkest red and the lowest ranking 10% are coloured darkest blue.

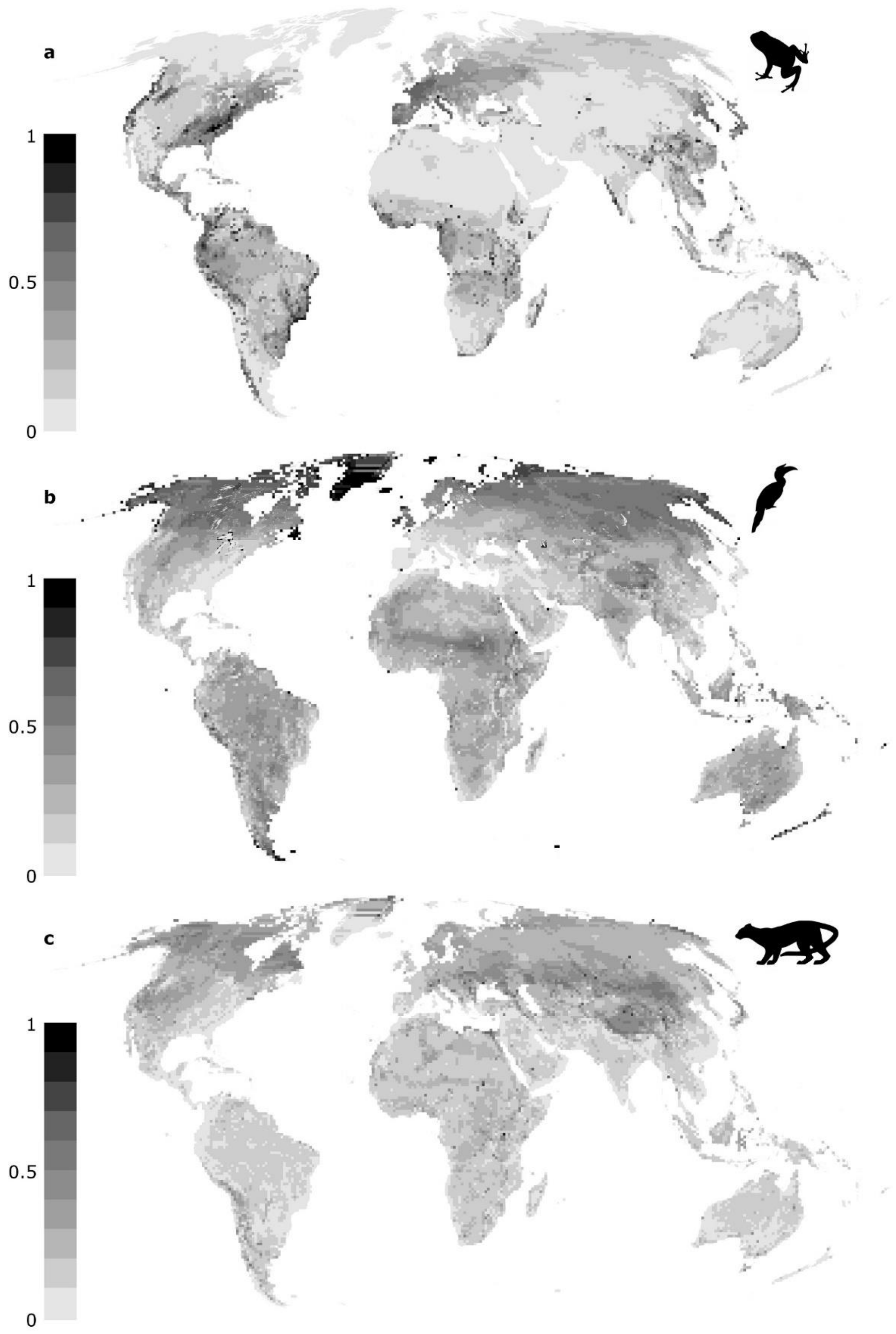

Supplementary Figure 6: Global contributions to tetrapod Human-Impacted Phylogenetic Endemism (HIPE) by non-reptilian tetrapods. The proportional contributions to tetrapod HIPE scores by a) amphibians, b) birds and c) mammals. Grid cell scores range from 1 (100% of HIPE contributed by clade; black) to 0 (0% of HIPE contributed by clade; light grey).

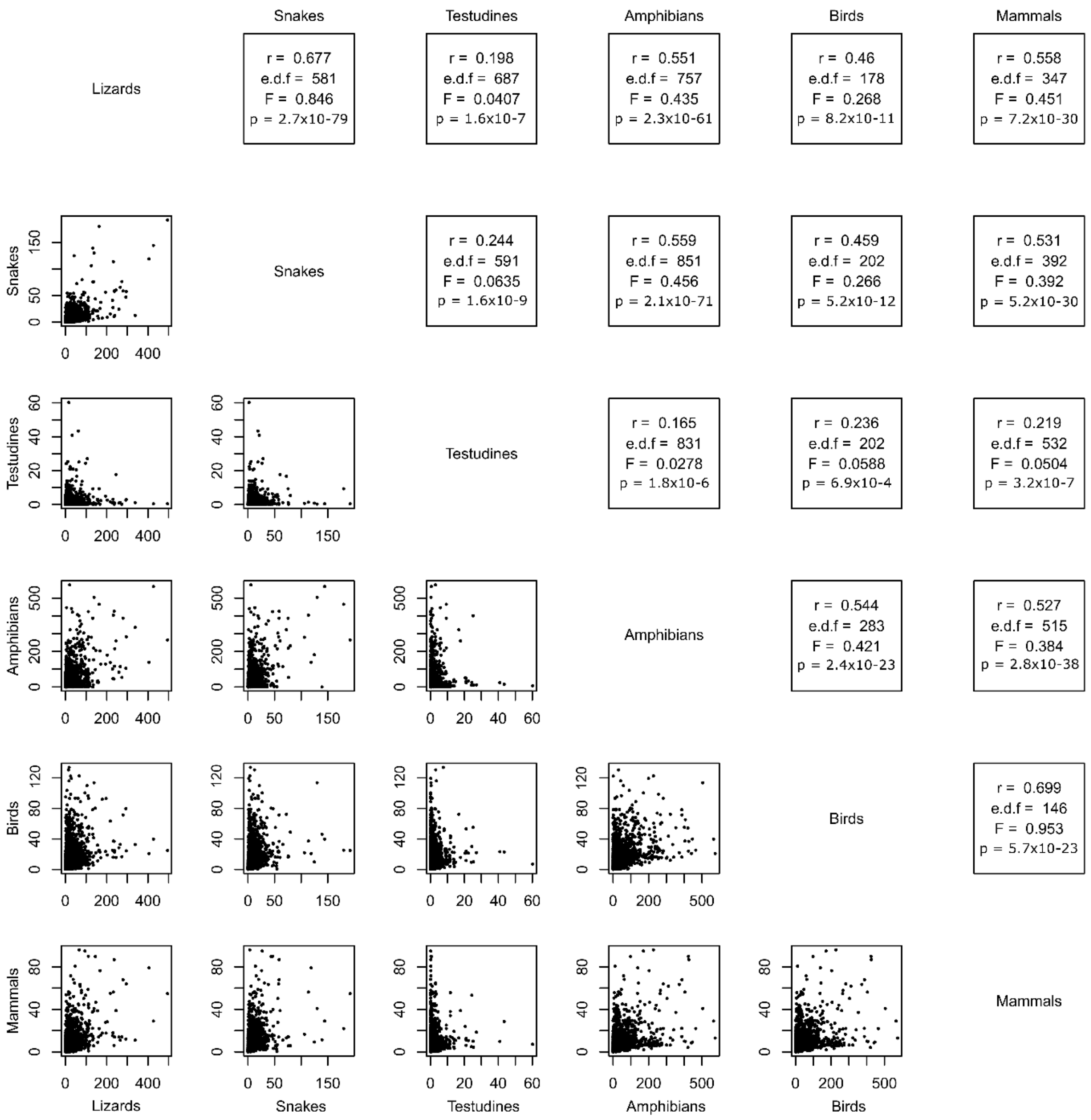

Supplementary Figure 7: relationships amongst reptile and tetrapod groups for all grid cells of global Human-Impacted Phylogenetic Endemism (HIPE). Results of spatially corrected Pearson's correlations for between HIPE scores of tetrapod groups (above diagonal split) and scatterplots of the values for each non-zero grid cell of global HIPE values for each tetrapod group (below diagonal split).

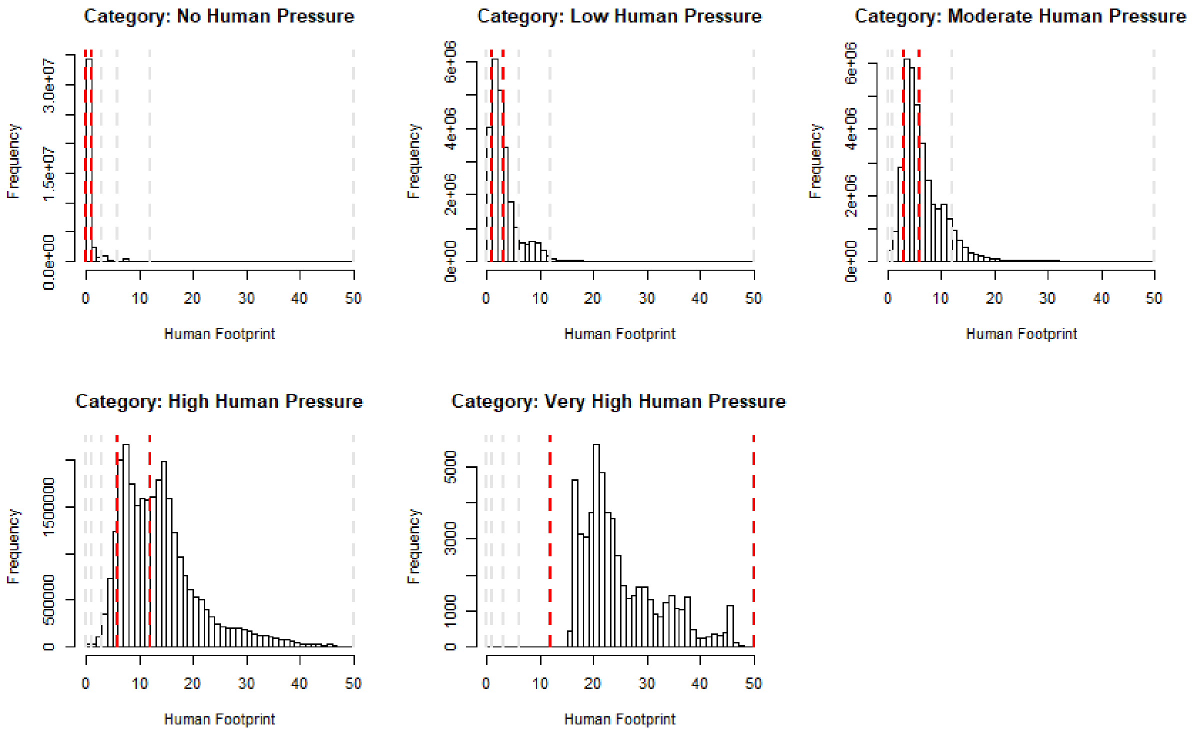

Supplementary Figure 8: Distribution of untransformed Human Footprint values when aggregated to lower resolution 'Human Pressure' grid cells. Each histogram represents the distribution of original Human Footprint values (0-50) at a 1 km x 1 km resolution that are found within the overlapping lower-resolution grid cells which correspond to each of the five broader Human Pressure categories (e.g. 'No Pressure') when the low-resolution cells are aggregated and upscaled. The grey dashed lines on each histogram represent the boundaries of each Human Pressure category, and the red dashed lines highlight the boundaries for the category featured in the histogram. The high resolution values that were coincident with each lower resolution (96.5 x 96.5 km) grid cell were averaged and the category within which the average value fell (e.g. a mean of 15 would lead to the upscaled (lower-resolution) grid cell being listed as 'Very High Pressure', the boundaries for which are 12-50). Thus, each panel contains all high-resolution Human Footprint grid cell values which were coincident with each low-resolution grid cell assigned to a specific Human Pressure category, the boundary of which is outlined by the red dashed lines: 'No Human Pressure' (n = 39,918,002 high-resolution grid cells); 'Low Human Pressure' (n = 24,853,276 high-resolution grid cells); 'Moderate Human Pressure' (n = 37,021,401 high-resolution grid cells); 'High Human Pressure' (n = 27,836,357 high-resolution grid cells); and 'Very High Human Pressure' (n = 56,067 high-resolution grid cells).

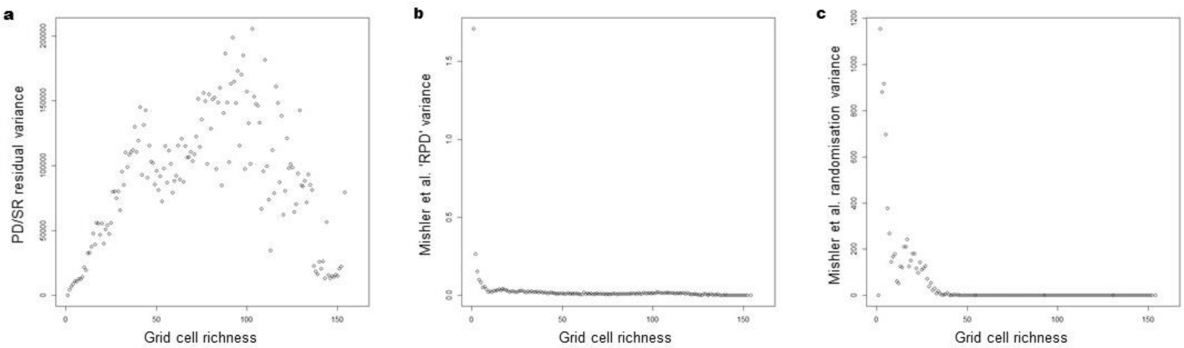

Supplementary Figure 9: Variance of values for three methods to measure the deviation from expected phylogenetic diversity (PD). The variance at each observed grid cell species richness for reptiles across three methods: a) residuals from a linear regression of PD against richness; b) the 'relative' PD method of Mishler et al.[1]; and c) the randomisation method of Mishler et al.[1]

Supplementary Table 1: Mathematical definitions of phylogenetic diversity and endemism-based metrics used in study. A simple description, calculation, and worked example for each of the spatial and species-level metrics employed herein.

Metric	Description	Calculation	Example
Phylogenetic Diversity (PD)[2]	PD is a biodiversity measure which sums the phylogenetic branch lengths spanning a set of taxa. For spatial mapping, the branches spanning a set of taxa found in a grid cell can be summed to provide a measure of PD present in the grid cell.	PD_i, the Phylogenetic Diversity of grid cell *i*, is given by $$PD_i = \sum_{b=1}^{2S_i-2} L_{b,i}$$ Where $L_{b,i}$ is the length of phylogenetic branch *b*, in the sub tree connecting all species in cell *i*. S_i is the species richness in cell *i* so that $2S_i - 2$ is the total number of branches spanning the species present in the cell (not counting the stem).	For a grid cell containing three species, connected by four branches of length 2, 2, 5, and 7 MY. Total PD equals the summed length of each of the five branches: 2 + 2 + 5 + 7 = 16 (units are MY/grid cell area).<br>When only one species is present in the grid cell, PD is equal to terminal branch length (TBL), the length of the branch connecting the species at the tip to all other branches in the phylogenetic tree.
Weighted Endemism (WE)[3,4]	WE is a metric that applies to a grid cell. It captures how critical the cell is for its contributions to the total range size of the species it contains. WE is calculated as a sum over all species in the grid cell. Each species' contribution to WE is given by the fraction of its total range that lies within the cell.	WE_i, the weighted endemism of cell *i* is given by $$WE_i = \sum_{j=1}^{S} q_{ij}$$ Where q_{ij} is the fraction of the range (in grid cells) of species *j* found in grid cell *i* and *S* is the number of species in the grid cell. The sum can be considered as a sum over all species as $q_{ij} = 0$ if species *j* is not found in grid cell *i*.	For a focal grid cell containing two species, A and B. Species A occurs in a total of 5 grid cells globally, including our focal cell. Species B occurs in a total of 4 grid cells globally, including our focal cell. Species A contributes (1/5) to WE and species B contributes (1/4). This gives<br>WE = (1/5) + (1/4) = 0.45 (units are grid cell area^{-1}).
Evolutionary Distinctness Rarity (EDR)[5]	EDR is a metric for a given species. Specifically, it measures unique evolutionary history (Evolutionary Distinctiveness: ED) of the species weighted by the species' rarity as inferred by its total range size. The EDR score is then summed for each species in a grid cell to provide a grid cell score for spatial mapping.	EDR_i, the EDR score of cell *i* is given by $$EDR_i = \sum_{j=1}^{S} ED_j \cdot q_{ij}$$ Where ED_j is the Evolutionary Distinctiveness of species *j* and q_{ij} is the fraction of the distribution (in grid cells) of species *j* found in cell *i*, which	For a species that occurs in 5 grid cells and has an ED of 10 MY (million years), that species has an EDR of 10*(1/5) = 2 (units are MY/grid cell). For a grid cell with three species, whose EDR values are 2, 4 and 6, the EDR of the grid cell is 2 + 4 + 6 = 12 (units are MY/grid cell area).

		should be one of its occupied cells, in practice q_{ij} is given by the reciprocal of the species' range size ($1/R_j$), and S is the species richness of the grid cell.	
Phylogenetic Endemism (PE)[4]	An extension of Weighted Endemism which distributes the length of each phylogenetic branch equally across all grid cells in which that phylogenetic branch is found. If total PD of any branch was spread evenly in space across its range, PE measures the amount of phylogenetic diversity (PD) accumulated within each grid cell after the spreading. The sum of PE across all global grid cells recovers global PD.	PE_i, the Phylogenetic Endemism score of cell *i* is given by $$PE_i = \sum_{b=1}^{2S-2} L_b q_{i,b}$$ Where L_b is the length of phylogenetic branch *b*, S_i is the total (global) species richness so that $2S_i - 2$ is the total number of branches in the full phylogenetic tree and $q_{i,b}$ is the total proportion of the range of branch *b* that falls within cell *i*. The sum can be considered as a sum over all species as $q_{ij} = 0$ if the branch has no descendent species in grid cell *i.*	For a grid cell containing three phylogenetic branches: one branch unique to species A (branch A), one branch unique to species B (branch B), both of which are 8 MY in length, and a third branch from which both species are descended (branch C), which is 4 MY in length. Branch A occurs in 5 grid cells, branch B in 4 cells, but both species co-occur in two grid cells, thus branch C occurs only in (5+4-2 = 7) grid cells. Thus PE = (8/5)+(8/4)+(4/7) = 4.17 MY/grid cell area.
Human-Impacted Phylogenetic Endemism (HIPE)	An extension of Phylogenetic Endemism which weights each grid cell based on the level of human pressure as defined by Venter et al.[6], scaled between 0 and 1, in that cell to provide a 'Human Pressure (HP)-weighted distribution'. Phylogenetic Endemism is then calculated based on these HP-weighted distributions for each branch of the tree rather than on the geographic range size. Less impacted cells thus receive a higher score if ranges are spread across a landscape with variable levels of human pressure. Where the human pressure is	$HIPE_i$ the Human-Impacted Phylogenetic Endemism of cell *i* is given by $$HIPE_i = \sum_{b=1}^{2S-2} L_b \times \frac{H_{i,j}}{HP_j}$$ Where L_b is the length of each phylogenetic branch *b*, S is the global species richness so that $2S - 2$ is the total number of branches (excluding the stem), $H_{i,j}$ is the HP-weighted distribution of species *j* in *i* (zero if the species is absent from cell i) and HP_j is the total HP-weighted distribution of species *j* across all cells.	For a grid cell with an HP-weighted value of 0.8 (in our analyses 0.8 is equivalent to "low human pressure" of Venter et al.[6], see Methods), containing three phylogenetic branches: one branch unique to species A (branch A), one branch unique to species B (branch B), both of which are 8 MY in length, and a third branch from which both species are descended (branch C), which is 4 MY in length. Branch A occurs in 5 grid cells, each with a HP-weighted value of 0.8. Branch B occurs in 4 cells, two which it shares with Branch A (HP-weighted value = 0.8) and two with a HP-weighted value of 0.2. Branch C

	equal for all cells in an analysis, HIPE gives the same result as PE.		therefore occurs only in seven grid cells, 5 of which have a HP-weighted value of 0.8 and two of 0.2.<br><br>HIPE = (8*(0.8/(5*0.8))) + (8*(0.8/(2*0.2+2*0.8))) + (4*(0.8/(5*0.8+2*0.2))) = 5.527 MY/grid cell area<br><br>Where 8*(0.8/(5*0.8)) is the length of branch A (8 MY) multiplied by the proportion of the focal grid cell's HP-weighted distribution sore (0.8) contribution to the total HP-weighted distribution score of the species of (5*0.8) when a species occurs in 5 grid cells of 0.8. This is repeated on each line for branch B and C and their constituent grid cells.
Terminal Endemism (TE)	A species level measure chosen to incorporate the common elements of EDR and PE: Terminal Branch Length (TBL). TE represents the terminal branch length of a taxa scaled by a measure of its rarity, given by the reciprocal of its range size. Both PE and EDR can be written in the form TE + I where I represents an additional component to account for interior branches that is different for EDR and PE.	TE_j, the Terminal Endemism score of a species *j*, is given by<br>$$TE_j = TBL_j \cdot \frac{1}{R_j}$$<br>Where TBL_j is the Terminal Branch Length of species *j* in the phylogeny, and R_j is the total range size of species *j* measured in number of grid cells.	For a species that occurs in 5 grid cells and has a terminal branch length of 7 MY (million years), that species has a TE score of 7*(1/5) = 1.4 (units are MY/grid cell area).
Human-Impacted Terminal Endemism (HITE)	HITE is an extension of TE (above) and represents the terminal branch length of a taxa scaled by a measure of its rarity, given by the reciprocal of its Human Pressure-weighted distribution.	$HITE_j$, the Human-Impacted Terminal Endemism score of a species *j* is given by<br>$$HITE_j = TBL_j \cdot \frac{1}{HR_j}$$<br>Where TBL_j is the Terminal Branch Length of species *j* in the phylogeny, and HR_j is the summed	For a species that occurs in 5 grid cells (two cells with an HP-weighted value of 0.2 and three cells with an HP-weighted value of 0.6), then the species has a total Human Pressure-weighted distribution of (2*0.2 + 3 *0.6). Suppose the species has a terminal branch length

		Human Pressure-weighted distribution of species *j* across all grid cells.	of 7 MY (million years), that species has an HITE score of 7*1/(2*0.2 + 3 *0.6) = 3.182 (units are MY/grid cell area).

Supplementary Table 2: Taxonomic representation for each reptilian order and tetrapod class in this study. Number and percentage of species for each clade with both spatial and phylogenetic data available. Data sources in Supplementary References.

Clade	Total PD (MY) (number of species and % of described species in phylogeny)*	Species with phylogenetic and range data	Percentage of total species (total number of species)
Reptiles	136,962 (9,874 spp.; 91%)	9,862	90.9% (10,845)[7]
Crocodilians	531 (23 spp.; 95.8%)	23	95.8% (24)[7]
Testudines	8,213 (294 spp.; 80.4%)	282	80.3% (351)[7]
Lepidosaurs	128,218 (9,557 spp.; 91.3%)	9,557	91.3% (10,470)[7]
Amphibians	130,703 (7,239 spp.; 93.1%)	5,874	75.5% (7,776)[8]
Birds	85,469 (9,993 spp.; 91.1%)	9,274	84.5% (10,970)[9]
Mammals	46,649 (4,751 spp.; 83.5%)	4,386	77% (~5,692)[10]
**Tetrapods**	**399,783 (31,857 spp.; 91.1%)**	**29,396**	**84.2% (~34,906)**

*Median value taken from random sample of 100 phylogenies for all clades except testudines and crocodilians, for which only single consensus phylogenies were available

Supplementary Table 3: Median diversity metric scores for each clade under equal taxonomic representation. The median values for three metrics when each phylogenetic tree is randomly stripped of species to contain 75.5% of all described species. This is to match the lowest taxonomic coverage for any clade in an analysis in the manuscript: 75.5% of amphibians with phylogenetic and range data. Human-Impacted Phylogenetic Endemism (HIPE) values are the median grid cell value from spatial analyses. Phylogenetic diversity (PD) and terminal branch length (TBL) values are the median calculated across a distribution of 100 phylogenetic trees that have been randomly stripped of species to contain ~75.5% of species (rounded to nearest integer). For amphibians, birds and lepidosaurs species were dropped at random once from each of the 100 phylogenetic trees used for all analyses. For crocodilians and testudines, for which we used single consensus phylogenetic trees, we randomly dropped the required number of species to retain a phylogenetic tree with ~75.5% of species and repeated the process 100 times to generate a distribution of PD and TBL scores.

Clade	No. of species at 75.5%	Median HIPE (x 10^{-4} MY/km^2)	Median Total PD (MY)	Median TBL (MY)
Amphibians	5,874	4.2	114,724	8.4
Birds	8,283	4.1	73,435	3.4
Mammals	4,297	3.5	45,383	4.0
Reptiles	8,188	6.1	121,997	
Crocodilians	18	0.14	7,985	5.9
Testudines	265	1.1	476	15.4
Lepidosaurs	7,905	6.7	113,536	5.6

Supplementary Table 4: The ten highest ranking Human-Impacted Terminal Endemism (HITE) species for each tetrapod group. The ten species with the largest HITE scores for each group and their IUCN Red List status as of December 2018. NE = Not Evaluated, DD = Data Deficient, LC = Least Concern, NT = Near Threatened, VU = Vulnerable, EN = Endangered, CR = Critically Endangered. Terminal branch length (TBL) is multiplied by the reciprocal of the HP-weighted distribution score to calculate HITE. Human Pressure (HP)-weighted distribution scores are the summed human pressure values (between 0.2 and 1; see Methods) for all grid cells in which the species occurs.

Species	HP-weighted distribution score	TBL (MY)	HITE (x 10^{-2} MY/km^2)	IUCN Red List Status
Lizards				
Dibamus somsaki	0.4	140.2	3.76	DD
Dibamus dalaiensis	0.4	119.1	3.20	LC
Goniurosaurus kuroiwae	0.2	53.7	2.88	VU
Gekko canaensis	0.2	52.1	2.80	LC
Brachymeles wrighti	0.2	50.2	2.70	DD
Cricosaura typica	0.4	74.4	2.00	NT
Luperosaurus yasumai	0.2	36.8	1.98	DD
Dibamus vorisi	0.4	71.2	1.91	DD
Cnemaspis psychedelica	0.2	33.0	1.77	EN
Gonatodes daudini	0.2	33.0	1.77	CR
Snakes				
Gerrhopilus bisubocularis	0.2	49.2	2.64	DD
Epictia rubrolineata	0.2	26.7	1.43	DD
Gerrhopilus oligolepis	0.2	25.0	1.34	DD
Bitia hydroides	0.2	23.3	1.25	LC
Tricheilostoma greenwelli	0.2	22.0	1.18	DD
Gerrhopilus tindalli	0.2	18.5	0.99	DD
Oligodon travancoricus	0.2	17.2	0.92	DD
Pareas nigriceps	0.4	33.5	0.90	DD
Opisthotropis tamdaoensis	0.2	15.0	0.81	DD
Tetracheilostoma bilineatum	0.4	30.0	0.81	LC
Testudines				
Pseudemydura umbrina	0.4	89.6	2.40	CR
Geoemyda japonica	0.2	28.5	1.53	EN
Elusor macrurus	0.4	37.3	1.00	EN
Astrochelys yniphora	0.4	30.8	0.83	CR
Siebenrockiella leytensis	0.4	30.4	0.82	CR
Pyxis planicauda	0.4	16.5	0.44	CR

Myuchelys georgesi	0.6	21.7	0.39	DD
Myuchelys purvisi	2.6	53.8	0.22	NE
Pyxis arachnoides	0.8	16.5	0.22	CR
Pelusios broadleyi	0.6	9.1	0.16	VU
Amphibians				
Chikila fulleri	0.2	117.5	6.31	DD
Karsenia koreana	0.2	84.5	4.54	LC
Nasikabatrachus sahyadrensis	0.4	145.5	3.91	EN
Phytotriades auratus	0.2	56.5	3.04	CR
Latonia nigriventer	0.2	56.5	3.03	CR
Ptychadena filwoha	0.4	110.1	2.96	DD
Eleutherodactylus counouspeus	0.2	53.2	2.86	EN
Platymantis isarog	0.2	52.3	2.81	LC
Micrixalus narainensis	0.2	51.0	2.74	DD
Scinax muriciensis	0.2	49.1	2.64	CR
Birds				
Microeca hemixantha	0.2	23.7	1.27	NT
Nipponia nippon	0.2	20.7	1.11	EN
Regulus madeirensis	0.2	17.9	0.96	LC
Zeledonia coronata	0.2	17.5	0.94	LC
Circus maillardi	0.2	12.8	0.69	EN
Papasula abbotti	0.2	12.5	0.67	EN
Nesoenas mayeri	0.2	12.2	0.66	VU
Nesillas mariae	0.2	11.8	0.64	LC
Dicaeum quadricolor	0.2	10.6	0.57	CR
Leucocarbo carunculatus	0.2	10.5	0.56	VU
Mammals				
Calcochloris tytonis	0.2	27.6	1.48	DD
Myrmecobius fasciatus	0.4	30.5	0.82	EN
Spalax arenarius	0.2	15.1	0.81	EN
Thomomys bulbivorus	0.2	14.5	0.78	LC
Gymnobelideus leadbeateri	0.4	25.9	0.70	CR
Hipposideros inexpectatus	0.4	25.7	0.69	DD
Niviventer culturatus	0.2	12.3	0.66	LC
Crocidura wimmeri	0.2	11.6	0.62	CR
Mus famulus	0.2	11.2	0.60	EN
Crocidura orientalis	0.2	10.9	0.59	LC

Supplementary References

1. Mishler, B. D. *et al.* Phylogenetic measures of biodiversity and neo- and paleo-endemism in Australian Acacia. *Nat. Commun.* **5**, 4473 (2014).

2. Faith, D. P. Conservation evaluation and phylogenetic diversity. *Biol. Conserv.* **61**, 1–10 (1992).

3. Roll, U. *et al.* The global distribution of tetrapods reveals a need for targeted reptile conservation. *Nat. Ecol. Evol.* **1**, 1677–1682 (2017).

4. Rosauer, D., Laffan, S. W., Crisp, M. D., Donnellan, S. C. & Cook, L. G. Phylogenetic endemism: A new approach for identifying geographical concentrations of evolutionary history. *Mol. Ecol.* **18**, 4061–4072 (2009).

5. Jetz, W. *et al.* Global Distribution and Conservation of Evolutionary Distinctness in Birds. *Curr. Biol.* **24**, 919–930 (2014).

6. Venter, O. *et al.* Sixteen years of change in the global terrestrial human footprint and implications for biodiversity conservation. *Nat. Commun.* **7**, 12558 (2016).

7. Uetz, P., Freed, P. & Hosek, J. The Reptile Database. http://www.reptile-database.org (2019).

8. Frost, D. R. Amphibian Species of the World: an Online Reference. Version 6.0. *American Museum of Natural History, New York, USA.* http://research.amnh.org/herpetology/amphibia/index.html (2019).

9. BirdLife. BirdLife Taxonomic Checklist 2.0. http://datazone.birdlife.org/species/taxonomy (2017).

10. IUCN. IUCN Red List of Threatened Species. Version 2019-1. www.iucnredlist.org (2019).
