# Supplementary material for: Global priorities for conservation of reptilian phylogenetic diversity in the face of human impacts
Source: Nat Commun. 2020 May 26;11:2616. doi: 10.1038/s41467-020-16410-6 (PMC7250838; doi:10.1038/s41467-020-16410-6)
Supplement: Supplementary file 2 — Reporting Summary [file 41467_2020_16410_MOESM2_ESM.pdf]

nature research

Corresponding author(s): Rikki Gumbs

Last updated by author(s): Apr 16, 2020

Reporting Summary

Nature Research wishes to improve the reproducibility of the work that we publish. This form provides structure for consistency and transparency in reporting. For further information on Nature Research policies, see Authors & Referees and the Editorial Policy Checklist .

Statistics

For all statistical analyses, confirm that the following items are present in the figure legend, table legend, main text, or Methods section.

n/a	Confirmed	
☐	☒	The exact sample size (*n*) for each experimental group/condition, given as a discrete number and unit of measurement
☐	☒	A statement on whether measurements were taken from distinct samples or whether the same sample was measured repeatedly
☐	☒	The statistical test(s) used AND whether they are one- or two-sided<br>*Only common tests should be described solely by name; describe more complex techniques in the Methods section.*
☒	☐	A description of all covariates tested
☐	☒	A description of any assumptions or corrections, such as tests of normality and adjustment for multiple comparisons
☐	☒	A full description of the statistical parameters including central tendency (e.g. means) or other basic estimates (e.g. regression coefficient) AND variation (e.g. standard deviation) or associated estimates of uncertainty (e.g. confidence intervals)
☐	☒	For null hypothesis testing, the test statistic (e.g. *F*, *t*, *r*) with confidence intervals, effect sizes, degrees of freedom and *P* value noted<br>*Give P values as exact values whenever suitable.*
☒	☐	For Bayesian analysis, information on the choice of priors and Markov chain Monte Carlo settings
☒	☐	For hierarchical and complex designs, identification of the appropriate level for tests and full reporting of outcomes
☐	☒	Estimates of effect sizes (e.g. Cohen's *d*, Pearson's *r*), indicating how they were calculated

Our web collection on statistics for biologists contains articles on many of the points above.

Software and code

Policy information about availability of computer code

Data collection	No software was used
Data analysis	All analyses were conducted using R version 3.6.0. The custom code for the R functions to generate the novel diversity metrics are available on GitHub (https://github.com/rgumbs/HIPE), and all code to repeat all analyses is available upon request.

For manuscripts utilizing custom algorithms or software that are central to the research but not yet described in published literature, software must be made available to editors/reviewers. We strongly encourage code deposition in a community repository (e.g. GitHub). See the Nature Research guidelines for submitting code & software for further information.

Data

Policy information about availability of data

All manuscripts must include a data availability statement. This statement should provide the following information, where applicable:

- Accession codes, unique identifiers, or web links for publicly available datasets
- A list of figures that have associated raw data
- A description of any restrictions on data availability

The data that support the findings of this study are available from the corresponding author upon request. The source data underlying Fig 5a-c are provided as a Source Data file.

Field-specific reporting

Please select the one below that is the best fit for your research. If you are not sure, read the appropriate sections before making your selection.

Ecological, evolutionary & environmental sciences study design

All studies must disclose on these points even when the disclosure is negative.

Study description	We used available phylogenetic, geographic range and extinction risk data for the world's reptiles to determine highly irreplaceable regions and species in regions of high human pressure that are of conservation importance, and contrasted the results for reptiles with those for amphibians, birds and mammals.
Research sample	Phylogenetic data were taken from published phylogenies for amphibians (Jetz and Pyron 2018; doi:10.1038/s41559-018-0515-5), birds (Jetz et al. 2014; doi: 10.1016/j.cub.2014.03.011), mammals (Kuhn et al. 2011; doi: 10.1111/j.2041-210X.2011.00103.x) and reptiles (Tonini et al. 2016; doi: 10.1016/j.biocon.2016.03.039). Spatial data were taken from the IUCN Red List for amphibians and birds, from Roll (2017; doi: 10.1038/s41559-017-0332-2) for reptiles, and provided by BirdLife International for birds. Extinction risk data were taken from IUCN Red List. Human pressure data were taken from the publicly available Human Footprint dataset (Venter et al. 2016; doi: 10.1038/ncomms12558).
Sampling strategy	The sample size of species included for each clade was determined by data availability - limited to those species with both phylogenetic and spatial data for each tetrapod class. The number of phylogenies used for replication (100) was selected to follow previous analyses of a similar nature (e.g. Tonini et al. 2016).
Data collection	Data were collected from published and publicly available datasets.
Timing and spatial scale	Data are of a global scale and all datasets used were published before January 2019.
Data exclusions	Species for which both phylogenetic and spatial data was unavailable or could not be reliably matched were dropped from our analyses to reduce the impact of potentially invalid species (e.g. species now synonymised but previously considered valid) and extinct species on our results. These exclusion critera were pre-established.
Reproducibility	We repeated our analyses 100 times for each clade with a distribution of phylogenies available to capture phylogenetic uncertainty.
Randomization	We randomly selected the 100 phylogenies for use in our analyses from larger distributions (10,000 trees) when such distributions were available (i.e. for birds, mammals, amphibians and lepidosaur reptiles). For the analyses which required randomisation for the development of a null distribution against which to compare observed results, the species selected over multiple replicates (100 or 1,000) were selected at random.
Blinding	Blinding was not relevant to this study, which focused on global patterns of biodiversity and conservation importance.

Did the study involve field work? ☐ Yes ☒ No

Reporting for specific materials, systems and methods

We require information from authors about some types of materials, experimental systems and methods used in many studies. Here, indicate whether each material, system or method listed is relevant to your study. If you are not sure if a list item applies to your research, read the appropriate section before selecting a response.

Materials & experimental systems

n/a	Involved in the study
☒	☐ Antibodies
☒	☐ Eukaryotic cell lines
☒	☐ Palaeontology
☒	☐ Animals and other organisms
☒	☐ Human research participants
☒	☐ Clinical data

Methods

n/a	Involved in the study
☒	☐ ChIP-seq
☒	☐ Flow cytometry
☒	☐ MRI-based neuroimaging
